# AXL is a candidate receptor for SARS-CoV-2 that promotes infection of pulmonary and bronchial epithelial cells

**DOI:** 10.1038/s41422-020-00460-y

**Published:** 2021-01-08

**Authors:** Shuai Wang, Zongyang Qiu, Yingnan Hou, Xiya Deng, Wei Xu, Tingting Zheng, Peihan Wu, Shaofang Xie, Weixiang Bian, Chong Zhang, Zewei Sun, Kunpeng Liu, Chao Shan, Aifu Lin, Shibo Jiang, Youhua Xie, Qiang Zhou, Lu Lu, Jing Huang, Xu Li

**Affiliations:** 1 Key Laboratory of Structural Biology of Zhejiang Province, School of Life Sciences, Westlake University, Hangzhou, Zhejiang 310024 China; 2 Center for Infectious Disease Research, Westlake Laboratory of Life Sciences and Biomedicine, Hangzhou, Zhejiang 310024 China; 3 Institute of Biology, Westlake Institute for Advanced Study, Hangzhou, Zhejiang 310024 China; 4 Key Laboratory of Medical Molecular Virology (MOE/NHC/CAMS), School of Basic Medical Sciences and Biosafety Level 3 Laboratory, Fudan University, Shanghai, 200032 China; 5 The First Affiliated Hospital, Zhejiang University School of Medicine, Hangzhou, Zhejiang 310003 China; 6 Center for Biosafety Mega-Science, Wuhan Institute of Virology, State Key Laboratory of Virology, Chinese Academy of Sciences, Wuhan, Hubei 430071 China; 7 Key Laboratory for Cell and Gene Engineering of Zhejiang Province, College of Life Sciences, Zhejiang University, Hangzhou, Zhejiang 310058 China

**Keywords:** Molecular biology, Cell biology

## Abstract

The current coronavirus disease 2019 (COVID-19) pandemic presents a global public health challenge. The viral pathogen responsible, severe acute respiratory syndrome coronavirus 2 (SARS-CoV-2), binds to the host receptor ACE2 through its spike (S) glycoprotein, which mediates membrane fusion and viral entry. Although the role of ACE2 as a receptor for SARS-CoV-2 is clear, studies have shown that ACE2 expression is extremely low in various human tissues, especially in the respiratory tract. Thus, other host receptors and/or co-receptors that promote the entry of SARS-CoV-2 into cells of the respiratory system may exist. In this study, we found that the tyrosine-protein kinase receptor UFO (AXL) specifically interacts with the N-terminal domain of SARS-CoV-2 S. Using both a SARS-CoV-2 virus pseudotype and authentic SARS-CoV-2, we found that overexpression of AXL in HEK293T cells promotes SARS-CoV-2 entry as efficiently as overexpression of ACE2, while knocking out AXL significantly reduces SARS-CoV-2 infection in H1299 pulmonary cells and in human primary lung epithelial cells. Soluble human recombinant AXL blocks SARS-CoV-2 infection in cells expressing high levels of AXL. The AXL expression level is well correlated with SARS-CoV-2 S level in bronchoalveolar lavage fluid cells from COVID-19 patients. Taken together, our findings suggest that AXL is a novel candidate receptor for SARS-CoV-2 which may play an important role in promoting viral infection of the human respiratory system and indicate that it is a potential target for future clinical intervention strategies.

## Introduction

Coronavirus disease 2019 (COVID-19) has caused a global pandemic since December 2019 and presents a global public health threat. The causative viral pathogen, severe acute respiratory syndrome coronavirus 2 (SARS-CoV-2), is a highly contagious enveloped positive-strand RNA virus^1^ that causes upper respiratory diseases, fever and severe pneumonia in humans.^1,2^ SARS-CoV-2 belongs to the β coronavirus genus. Other members of this genus include severe acute respiratory syndrome coronavirus (SARS-CoV) and middle east respiratory syndrome coronavirus (MERS-CoV), which caused outbreaks in 2003 and 2012, respectively, though on a much smaller scale.^3,4^ SARS-CoV-2 preferentially infects cells of the respiratory tract^5^ but has been detected in almost all human organs, including the lungs, pharynx, heart, liver, brain, kidneys and digestive system organs.^6–8^

**Fig. 1:**
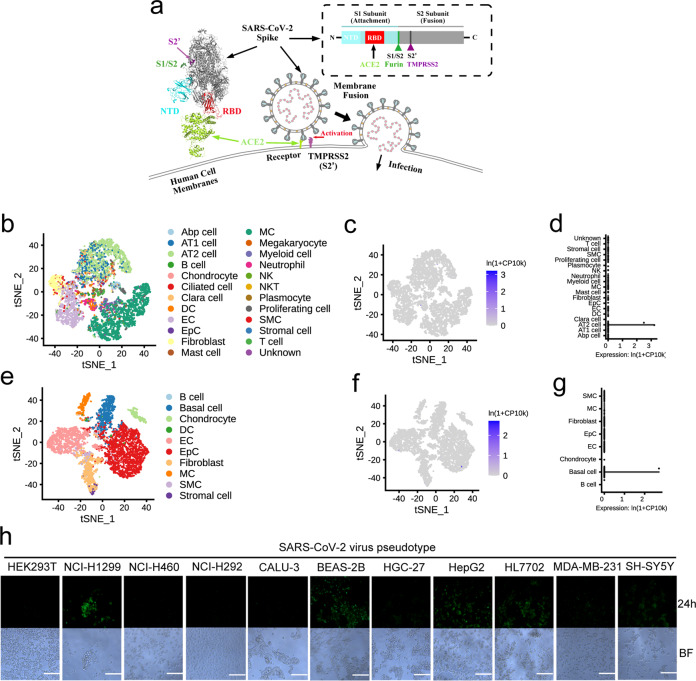
ACE2 expression is low in the human lungs and trachea. **a** Schematic of SARS-CoV-2 virion surface S-mediated receptor recognition and membrane fusion. During viral infection, the trimeric S protein is cleaved into S1 and S2 subunits, and the S1 subunits are released during the transition to the post-fusion conformation. The RBD on S1 directly binds to ACE2, while S2 is responsible for membrane fusion. Overview of the human cell landscape at the single-cell level in lung (**b**) and tracheal cells (**e**). Abp cell, alveolar bipotent progenitor cell; DC, dendritic cell; EC, endothelial cell; EpC, epithelial cell; MC, macrophages; NK, natural killer cell; SMC, smooth muscle cell. ACE2 expression levels in pulmonary (**c**, **d**) and bronchial cells (**f**, **g**) were evaluated using the human cell landscape at the single-cell level. Gene expression for each cell type was visualized using tSNE (**c**, **f**) and violin plots (**d**, **g**). **h** Eleven lung, liver, kidney, stomach, and neural cell lines were infected with the GFP-labeled SARS-CoV-2 pseudotype. The infection efficiency was evaluated using microscopy images taken at 24 h post infection. The scale bar indicates 250 μm. BF, bright field. The data shown are representative results from three independent experiments (*n* = 3).

Coronaviruses bind to host receptors through their spike (S) glycoproteins, which mediate membrane fusion and viral entry.^9^ The S protein is cleaved into the N-terminal S1 subunit and C-terminal S2 subunit by the host proteases Transmembrane protease serine 2 (TMPRSS2) and FURIN (Fig. 1a).^10^ SARS-CoV-2 shares 79.5% genetic identity with SARS-CoV, and studies have shown that ACE2, a cellular receptor for SARS-CoV,^11^ also binds SARS-CoV-2 S and serves as the entry point for SARS-CoV-2 (Fig. 1a).^1,12^ Although SARS-CoV-2 infection largely manifests with respiratory system symptoms, single-cell sequencing data indicate that overall ACE2 expression is low in various human tissues, especially pulmonary and bronchial tissues.^13,14^ A recently published single-cell mRNA sequencing dataset of 232,905 single cells from major adult organs^15^ revealed that ACE2 is specifically expressed in the kidneys and digestive system but rarely in organs such as the lungs and trachea (Supplementary information, Fig. S1a, b). The complex structures formed by binding between ACE2 and the receptor binding domain (RBD) of SARS-CoV-2 S have been resolved.^16,17^ However, recent studies have identified many neutralizing human antibodies that bind to SARS-CoV-2 S but do not bind the RBD.^18,19^ These results indicate that other important host receptors and/or co-receptors might exist that bind to different domain(s) of SARS-CoV-2 S and promote the entry of SARS-CoV-2 into cells of the respiratory system.

Using tandem affinity purification (TAP)-mass spectrometry (MS) to analyze protein complexes interacting with SARS-CoV-2 S in pulmonary and bronchial cells, we found that the tyrosine-protein kinase receptor UFO (AXL) specifically interacts with SARS-CoV-2 S. In HEK293T cells, AXL overexpression promoted viral entry as efficiently as ACE2 overexpression. Downregulating AXL, but not ACE2, significantly reduced infection of pulmonary cells by SARS-CoV-2. Soluble human recombinant AXL, but not ACE2, blocked SARS-CoV-2 infection in cells expressing high levels of AXL. Taken together, our results suggest AXL as a novel host receptor that promotes SARS-CoV-2 entry into human cells.

## Results

### Identification of SARS-CoV-2 S candidate receptors in H1299 and BEAS-2B cells

We analyzed the expression levels of ACE2 using a recently published single-cell mRNA sequencing dataset of 232,905 single cells from major adult human organs (all from non-SARS-CoV-2-infected samples)^15^ and found that ACE2 was specifically expressed in the jejunum (426/3075 cells), duodenum (95/2305 cells) and kidneys (681/20,053 cells) but rarely in organs such as the lungs (16/17,628 cells) and trachea (20/9521 cells) (Fig. 1b–g; Supplementary information, Table S1). To identify additional candidate receptors and/or co-receptors for SARS-CoV-2, we first assessed which of the following cell lines were vulnerable to infection: the human kidney-derived cell line HEK293T; the lung-derived cell lines NCI-H1299 (H1299), NCI-H460, NCI-H292, and CALU-3; the bronchus-derived cell line BEAS-2B; the stomach-derived cell line HGC-27; the liver-derived cell lines HepG2 and HL7702; the breast-derived cell line MDA-MB-231; and the neuron-derived cell line SH-SY5Y (Fig. 1h). The SARS-CoV-2 virus pseudotype successfully infected lung alveolar epithelial-like H1299 cells, immortalized normal bronchus BEAS-2B cells, immortalized liver carcinoma HepG2 cells and normal liver HL7702 cells (Fig. 1h). Thus, we used H1299 and BEAS-2B cells for the following studies to identify putative additional receptors or co-receptors responsible for SARS-CoV-2 infection of pulmonary and bronchial cells. To find the best strategy to mimic the expression and cleavage of SARS-CoV-2 S on the host cell membrane, we overexpressed full-length SARS-CoV-2 S, SARS-CoV-2 S RBD, or SARS-CoV-2 S S1+S2 in these cells or added recombinant full-length SARS-CoV-2 S or SARS-CoV-2 S RBD to the cells. Only cells overexpressing full-length SARS-CoV-2 S showed strong membrane localization of the S protein (Supplementary information, Fig. S2a). Moreover, full-length SARS-CoV-2 S was successfully cleaved in H1299 and BEAS-2B cells, generating fragments with sizes similar to those of S1-cleaved SARS-CoV-2 S (Supplementary information, Fig. S2b).

**Fig. 2:**
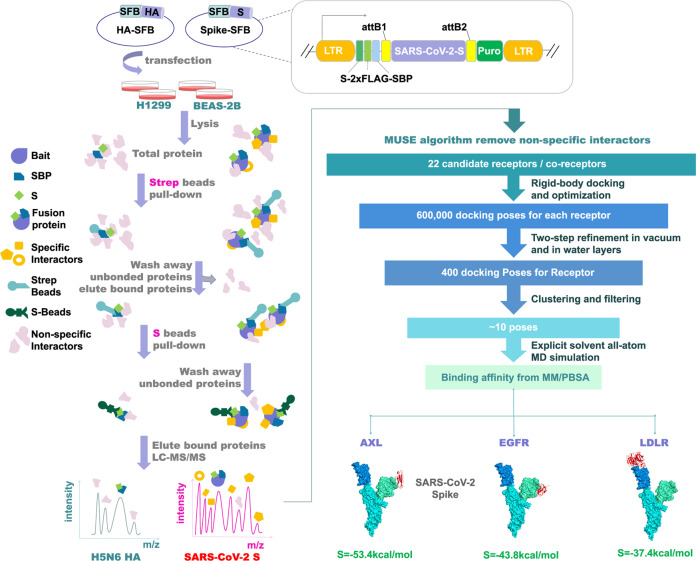
Schematic of the integrated proteomics-computation workflow used to identify potential SARS-CoV-2 receptors in human pulmonary and bronchial epithelial cells. (i) The SARS-CoV-2 S glycoprotein was constructed into an SFB tag-fused expression vector. (ii) H1299 and BEAS-2B cells stably expressing the SARS-CoV-2 S glycoprotein or control H5N6 HA glycoprotein were generated via transient transfection and puromycin selection. (iii) Interacting protein complexes were tandemly affinity-purified and identified by LC-MS profiling. (iv) High-confidence interacting proteins were generated with the MUSE statistical model. (v) The results were further enriched using a hierarchical computational protocol combining protein-protein docking, molecular modeling, and MM/PBSA binding affinity calculations from MD simulations of protein complexes. (vi) Three human receptor proteins (red), AXL, LDLR and EGFR, bound the SARS-CoV-2-S NTD (green) or RBD (blue) with reasonable affinity.

To identify host proteins responsible for SARS-CoV-2 infection of pulmonary and bronchial cells, we explored the proteins that interacted with SARS-CoV-2 S in H1299 and BEAS-2B cells. We established H1299 and BEAS-2B cells stably expressing N-terminal SFB (S–2× FLAG–SBP) triple-tagged SARS-CoV-2 S or control influenza A virus (A/Guangzhou/39715/2014 (H5N6)) haemagglutinin (HA) (Fig. 2). Twelve clones of each bait in each cell line were examined in the follow-up experiments, and the bait protein expression and localization were confirmed using western blotting and immunostaining analyses, respectively. We chose two clones of each bait in each cell line with membrane/cytosol localization and moderate expression as biological repeats for TAP-MS analysis and isolated, combined, and affinity-purified the membrane and soluble fractions using TAP. Proteins that associated with the bait in isolated complexes were identified by MS, and human, SARS-CoV-2 and H5N6 databases were searched for these proteins (Supplementary information, Fig. S2c–e and Tables S2, S3).

TAP-MS recovered a reasonable number of peptides, with high coverage for the bait proteins (Supplementary information, Fig. S2c and Table S2) and reasonable data reproducibility (Supplementary information, Fig. S2d). In total, we identified 21,972 peptides from 4228 proteins, representing 2153 unique prey proteins (Supplementary information, Table S3), 524 of which are membrane proteins or are able to translocate to the membrane. Interestingly, we did not identify ACE2 via TAP-MS for SARS-CoV-2 S in H1299 and BEAS-2B cells, indicating that ACE2 may not be the major host receptor of SARS-CoV-2 in these cells due to its low expression (Supplementary information, Fig. S2e and Table S3). Nonetheless, we did consistently identify ACE2 via TAP-MS for SARS-CoV-2 S in HEK293T cells, which express very low levels of ACE2,^1^ indicating that our approach is able to recover host receptors for SARS-CoV-2 S (Supplementary information, Fig. S2e and Table S3).

To distinguish bona fide SARS-CoV-2 S-interacting proteins from the large number of non-specific interactors frequently obtained in TAP-MS results,^20^ we carried out H5N6 HA TAP-MS experiments under experimental conditions identical to those used for controls and assigned a quality-associated probabilistic score to each binary interaction using the MUSE algorithm^21,22^ to remove non-specific interacting proteins. Twenty-two high-confidence candidate receptors/co-receptors were chosen for the following computational screening (Fig. 2).

### Computational screening of the top candidate receptors for SARS-CoV-2 S

To prioritize candidate receptors for viral entry experiments, the 22 high-confidence candidates were further enriched using a hierarchical computational protocol that combined protein-protein docking, molecular modeling, molecular dynamics (MD) simulations, and molecular mechanics/Poisson-Boltzmann surface area (MM/PBSA) calculations to find host receptor(s) with the highest binding affinity for SARS-CoV-2 S (Fig. 2). For each candidate receptor, we docked its extracellular domain with the full-length SARS-CoV-2 S and performed multiple rounds of conformational optimization using HADDOCK.^23^ The top-ranking poses were filtered by building full-length proteins and excluding complex conformations with potential steric clashes. Moreover, the stability of these docking poses was further assessed with explicit-solvent atomistic simulations. Eleven-nanosecond MD simulations were performed for each protein-protein docking conformation; to estimate the most likely binding conformation and the corresponding binding affinity, MM/PBSA calculations were conducted using the last 1-ns MD trajectories. The top three candidate receptors with the most favorable affinity scores, namely, AXL, epidermal growth factor receptor (EGFR), and low-density lipoprotein receptor (LDLR) (Fig. 2), were subjected to further biochemical experiments.

### SARS-CoV-2 S interacts with host AXL

**Fig. 3:**
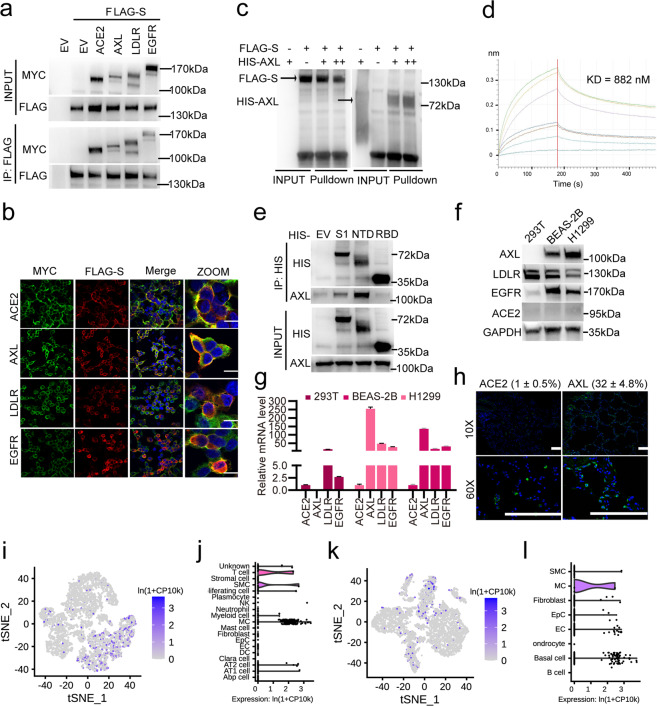
The SARS-CoV-2 S glycoprotein interacts with host AXL. **a** Validation of the interaction between SARS-CoV-2 S and ACE2, AXL, LDLR or EGFR. HEK293T cells were transfected with FLAG-tagged SARS-CoV-2 S and MYC-tagged ACE2, AXL, LDLR or EGFR for 24 h. The cells were lysed, and the lysates were incubated with FLAG-M2 resin; 5% lysate was used as the input control. Blots with antibodies recognizing the FLAG- or MYC-epitope tags are shown. **b** Co-localization assay of SARS-CoV-2 S and ACE2, AXL, LDLR or EGFR. HEK293T cells were transfected with the indicated constructs and subjected to immunofluorescence with an anti-FLAG antibody against SARS-CoV-2 S (red), an anti-MYC antibody against candidate receptors (green) and DAPI (blue) and visualized by microscopy. The scale bar indicates 15 μm. **c** In vitro pull-down assay of SARS-CoV-2 S and AXL. FLAG-tagged SARS-CoV-2 S and His-tagged AXL (amino acids 1–449) were expressed in HEK293T cells, affinity-purified, eluted and co-incubated for 1 h. Blots with antibodies recognizing the FLAG- or His-epitope tags are shown. **d** In vitro binding assay of SARS-CoV-2 S NTD and AXL. His-tagged SARS-CoV-2 S NTD and FLAG-tagged AXL were expressed in 293F cells, affinity-purified and eluted. The KD between His-tagged SARS-CoV-2 S NTD and FLAG-tagged AXL was measured using a BLI quantification assay. **e** Endogenous AXL interacts with the NTD of SARS-CoV-2 S. The in vitro-purified His-tagged SARS-CoV-2 S S1 domain, NTD and RBD were incubated with H1299 cell lysate and Ni-NTA resin; 5% lysate was used as the input control. Blots with antibodies recognizing endogenous AXL or His-epitope tags are shown. AXL is highly expressed in the H1299 and BEAS-2B cell lines. The expression of ACE2, AXL, LDLR and EGFR was examined in the HEK293T, H1299 and BEAS-2B cell lines by western blotting assay (**f**) and RT-qPCR (**g**). **h** AXL is highly expressed in human lung tissue. Human lung tissue sections were immunostained with antibodies against ACE2 or AXL (green) and with DAPI (blue) and visualized by confocal microscopy. The scale bars indicate 500 μm. AXL expression levels in pulmonary (**i**, **j**) and bronchial cells (**k**, **l**) were evaluated using the human cell landscape at the single-cell level. Gene expression for each cell type was visualized using tSNE (**i**, **k**) and violin plots (**j**, **l**). The data shown are representative results from three independent experiments (**a**–**h**, *n* = 3). The data are shown as the means ± SEM from three independent experiments.

We first validated the observed interactions using FLAG-tagged SARS-CoV-2 S and MYC-tagged AXL, EGFR and LDLR. All three candidate receptors pulled down SARS-CoV-2 S in HEK293T cells (Fig. 3a). AXL and SARS-CoV-2 S co-localized mainly to the cell membrane, as did ACE2, whereas LDLR and EGFR did not (Fig. 3b). Recombinant mFc-tagged SARS-CoV-2 S pulled down AXL in H1299 cells, as detected by MS, indicating that extracellular S is also capable of binding to AXL (Supplementary information, Table S4). In addition, recombinant His-tagged AXL robustly pulled down FLAG-tagged SARS-CoV-2 S in vitro (Fig. 3c). To examine the interaction between AXL and SARS-CoV-2 S, the interface was analyzed, and an extensive hydrophilic network was found along the interface. In contrast to the ACE2 receptor, AXL interacted with the S N-terminal domain (NTD) rather than the RBD (Supplementary information, Fig. S3a). Biolayer interferometry (BLI) quantification assays and in vitro binding assays confirmed that AXL directly interacts with the NTD of SARS-CoV-2 S (Fig. 3d–e). Mice appear to have low susceptibility to the virus,^1^ while other animals, including rhesus macaques,^24,25^ ferrets,^26^ and hamsters,^27^ have been reported to be infected with SARS-CoV-2 to different extents. To test if AXL contributes to the species tropism of SARS-CoV-2, sequence alignment of AXL was performed across humans, rhesus macaques, mice, hamsters and ferrets (Supplementary information, Fig. S3b), and the binding affinities of the AXL–S complexes for these species were quantitatively estimated from MM/PBSA calculations to be –66.5, –46.1, –39.5, –51.6 and –44.6 kcal/mol, respectively (Supplementary information, Fig. S3c). Analysis of the locations of mutations and their effects on interfacial hydrogen bonds indicated that AXL had the weakest binding with S protein in mice among the five species. Indeed, BLI quantification assays confirmed that murine AXL does not interact with the NTD of SARS-CoV-2 S (Supplementary information, Fig. S3d). These binding affinity data indicate that mice are the least subjected to SARS-CoV-2 infection among these species, which is consistent with the findings of previous studies.^1,24–27^

AXL is a receptor tyrosine kinase that transduces signals from the extracellular matrix into the cytoplasm^28^ and regulates many physiological processes, including cell survival, proliferation, differentiation and immune responses.^29–32^ To test whether AXL is expressed in human lung and tracheal cells and tissues, we first measured the protein and mRNA levels of these proteins in HEK293T, H1299 and BEAS-2B cells and human primary lung cells (Fig. 3f–h). ACE2 expression was low in all the cell lines tested, while LDLR and EGFR expression was detected in all cell lines (Fig. 3f, g). AXL levels were high in H1299 and BEAS-2B cells (Fig. 3f, g) as well as in human primary lung cells (Fig. 3h). Analysis of the human cell landscape at the single-cell level revealed that AXL was expressed in most human tissues tested (Supplementary information, Fig. S1c and Table S1). For example, a significantly higher percentage of cells in the lungs and trachea expressed AXL (1232/17,628 and 456/9521 cells, respectively) than expressed ACE2 (16/17,628 and 20/9521 cells, respectively) (Fig. 3i–l; Supplementary information, Table S1). AXL was not co-expressed with ACE2 or TMPRSS2 in human lungs or tracheas (Supplementary information, Fig. S4 and Table S1), suggesting that AXL’s function in promoting SARS-CoV-2 infection is independent of ACE2. Taken together, these findings indicate that AXL might be a novel receptor for SARS-CoV-2 in pulmonary and bronchial epithelial cells.

### AXL facilitates SARS-CoV-2 virus pseudotype entry into human cells

**Fig. 4:**
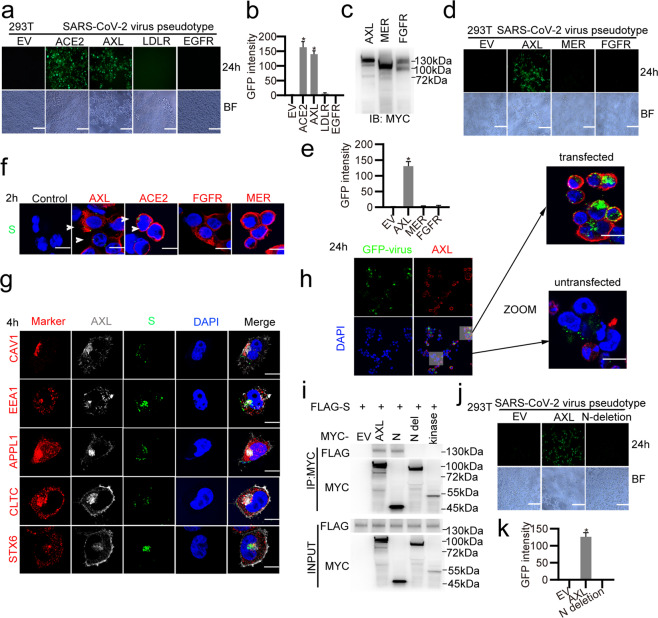
AXL binds to SARS-CoV-2 S and facilitates SARS-CoV-2 entry into host cells. **a** AXL facilitates SARS-CoV-2 virus pseudotype infection as potently as ACE2. HEK293T cells were transfected with MYC-tagged ACE2, AXL, LDLR or EGFR, infected with the GFP-labeled SARS-CoV-2 virus pseudotype, and visualized by microscopy at 24 h post infection. The scale bar indicates 250 μm. **b** The fluorescence intensities in **a** were quantitated as indicated. **c**–**e** AXL specifically facilitates SARS-CoV-2 virus pseudotype infection. **c** HEK293T cells were transfected with MYC-tagged AXL, MER or FGFR, and expression was evaluated by western blotting assay with antibodies recognizing the MYC epitope tag. **d** The cells were infected with the GFP-labeled SARS-CoV-2 virus pseudotype and visualized by microscopy at 24 h post infection. The scale bar indicates 250 μm. **e** The fluorescence intensities in **d** were quantitated as indicated. AXL binds to SARS-CoV-2 S and internalizes it in cooperation with endocytosis-related proteins. **f** AXL facilitates SARS-CoV-2 virus pseudotype binding to the cell surface. HEK293T cells were transfected with MYC-tagged AXL, ACE2, FGFR or MER and infected with a SARS-CoV-2 virus pseudotype for 2 h. The cells were fixed and subjected to immunofluorescence with an anti-FLAG antibody against the indicated proteins (red), an anti-SARS-CoV-2 S antibody (green) and DAPI (blue) and visualized by confocal microscopy. The white arrowheads indicate the virus. The scale bar indicates 15 μm. **g** AXL facilitates SARS-CoV-2 virus pseudotype entry into host cells, utilizing the host endocytosis system. H1299 cells were infected with a SARS-CoV-2 virus pseudotype for 4 h. The cells were fixed; subjected to immunofluorescence with antibodies against AXL (gray), SARS-CoV-2 S (green) and the indicated endocytosis-related proteins (red) and with DAPI (blue); and visualized by confocal microscopy. The scale bar indicates 15 μm. **h** AXL promotes SARS-CoV-2 virus pseudotype production in host cells. HEK293T cells were transfected with MYC-tagged AXL and infected with the GFP-labeled SARS-CoV-2 virus pseudotype for 24 h. The cells were fixed and subjected to immunofluorescence with an anti-MYC antibody against AXL (red) and DAPI (blue) and visualized by confocal microscopy. The scale bar indicates 20 μm. **i** The AXL NTD is required for binding with SARS-CoV-2 S. MYC-tagged wild-type or truncated AXL plasmids were transfected with FLAG-tagged SARS-CoV-2 S; co-IP assays were performed using an anti-MYC antibody, and epitope-tagged proteins were detected using western blotting assay. **j** The AXL NTD is required for SARS-CoV-2 virus pseudotype infection. HEK293T cells were transfected with full-length AXL or its N-terminal deletion mutant, infected with the GFP-labeled SARS-CoV-2 virus pseudotype and visualized by microscopy. The scale bar indicates 250 μm. **k** The fluorescence intensities in **j** were quantitated as indicated. The data shown are representative results from three independent experiments (**a**–**k**, *n* = 3). The data are shown as the means ± SEM from three independent experiments. *P* values were calculated using two-way ANOVA (*P < 0.05).

To determine whether SARS-CoV-2 uses AXL to facilitate entry into human cells, we first infected HEK293T cells, which express very low levels of ACE2 and AXL (Fig. 3f, g) and cannot normally be infected with the SARS-CoV-2 virus pseudotype (Fig. 1h), using a GFP-labeled SARS-CoV-2 virus pseudotype. We overexpressed ACE2, AXL, LDLR, EGFR or the empty vector in the HEK293T cells before infection. Viral infection was significantly enhanced in HEK293T cells overexpressing ACE2 (Fig. 4a, b), reproducing previous results^1,12^ and confirming that ACE2 is a SARS-CoV-2 receptor. Overexpressing AXL in HEK293T cells also greatly promoted viral infection in these cells, while overexpressing EGFR or LDLR failed to do so (Fig. 4a, b). We also overexpressed the tyrosine-protein kinase Mer (MER) or fibroblast growth factor receptor (FGFR); these proteins belong to the same TAM/TAM-like receptor families as receptor tyrosine kinases. Both MER and FGFR failed to promote viral entry into cells, indicating that the function of AXL in facilitating viral entry is highly specific (Fig. 4c–e). Compared with those overexpressing human AXL, cells overexpressing murine AXL or rhesus macaque AXL showed greatly reduced SARS-CoV-2 virus pseudotype infection percentages, indicating that AXL may not facilitate viral infection in these animal models (Supplementary information, Fig. S3e–g). SARS-CoV-2 virus pseudotype particles were observed on the surfaces of HEK293T cells overexpressing AXL at 2 h post infection, indicating that AXL facilitates SARS-CoV-2 virus pseudotype binding to the cell surface (Fig. 4f). Further evidence acquired by measuring the GFP mRNA of adsorbed viruses to cells (Supplementary information, Fig. S5a) and of internalized viruses in cells (Supplementary information, Fig. S5b) indicates that AXL enhances SARS-CoV-2 virus pseudotype attachment and entry to the host cells. Indeed, at 4 h post infection, SARS-CoV-2 S partially colocalized with AXL and host endocytosis and vesicle trafficking markers inside cells, including Caveolin1 (CAV1), Early endosome antigen 1 (EEA1), DCC-interacting protein 13-alpha (APPL1), Clathrin heavy chain 1 (CLTC) and Syntaxin-6 (STX6) (Fig. 4g), while knocking out AXL in H1299 cells blocked the colocalization between S and host endocytosis and vesicle trafficking markers (Supplementary information, Fig. S5c), indicating that SARS-CoV-2 is internalized and penetrates early endosomes in a clathrin-dependent manner after it binds AXL. AXL facilitates not only SARS-CoV-2 virus pseudotype entry but also viral reproduction. Overexpression of AXL in HEK293T cells greatly increased the number of SARS-CoV-2 virus pseudotype particles at 24 h post infection (Fig. 4h). To identify the region of AXL that binds the SARS-CoV-2 S protein, we generated several truncation mutants for AXL and found that the AXL extracellular NTD, but not the kinase domain, is responsible for the interaction (Fig. 4i) and for viral entry into host cells (Fig. 4j, k). Taken together, these results indicate that AXL facilitates SARS-CoV-2 entry as potently as ACE2.

**Fig. 5:**
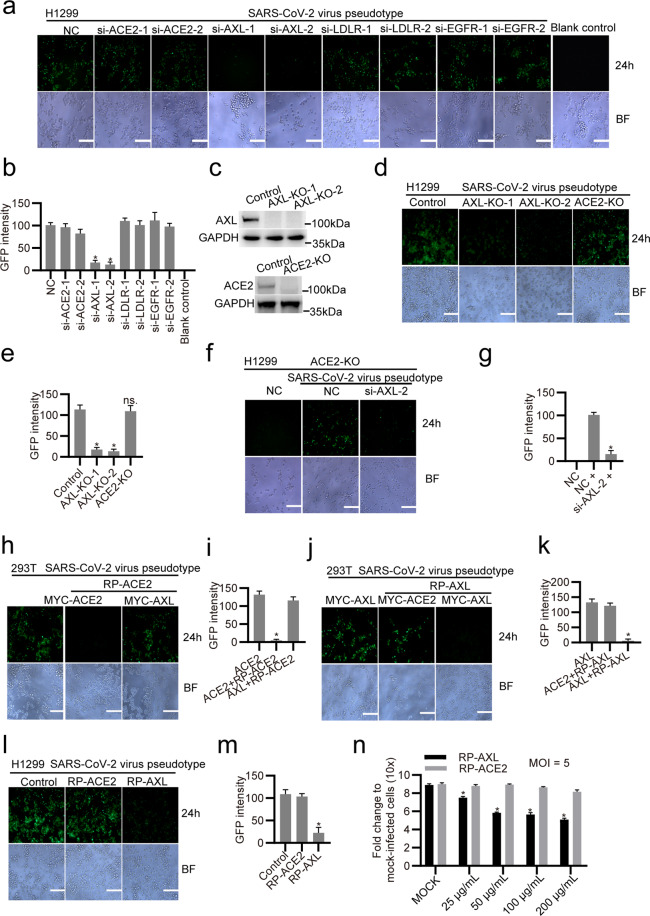
AXL promotes SARS-CoV-2 virus pseudotype infection in HEK293T and H1299 cells. **a**, **b** Knocking down AXL using siRNA impaired SARS-CoV-2 infection. H1299 cells were transfected with siRNAs against ACE2, AXL, LDLR and EGFR or with NC as a negative control and infected with the SARS-CoV-2 virus pseudotype for 24 h. **a** Microscopy images were taken at 24 h post infection. The scale bar indicates 250 μm. **b** The fluorescence intensities in **a** were quantitated as indicated. **c** ACE2- or AXL-KO H1299 cells were established using the CRISPR-Cas9 system, and the knockdown efficiencies were evaluated using western blotting assay with an antibody against ACE2 or AXL, respectively. **d** Wild-type H1299 and ACE2-/AXL-KO cells were infected with the GFP-labeled SARS-CoV-2 virus pseudotype and visualized by microscopy at 24 h post infection. The scale bar indicates 250 μm. **e** The fluorescence intensities in **d** were quantitated as indicated. **f**, **g** AXL’s function in mediating viral entry is independent of ACE2. **f** H1299 ACE2-KO cells were transfected with siRNAs against AXL or with NC as a negative control. The cells were infected with the GFP-labeled SARS-CoV-2 virus pseudotype and visualized by microscopy at 24 h post infection. The scale bar indicates 250 μm. **g** The fluorescence intensities in **f** were quantitated as indicated. **h**, **i** Soluble human ACE2 blocks SARS-CoV-2 virus pseudotype infection in cells overexpressing ACE2. **h** HEK293T cells were transfected with ACE2 for 24 h. Human recombinant His-ACE2 was mixed with the GFP-labeled SARS-CoV-2 virus pseudotype for 30 min and then added to the culture medium. The cells were washed at 2 h post infection and incubated with fresh medium. The cells were recovered after 24 h and visualized by microscopy. The scale bar indicates 250 μm. **i** The fluorescence intensities in **h** were quantitated as indicated. **j**, **k** Soluble human AXL blocks SARS-CoV-2 virus pseudotype infection in cells overexpressing AXL. **j** HEK293T cells were transfected with AXL for 24 h. Human recombinant His-AXL was mixed with the GFP-labeled SARS-CoV-2 virus pseudotype for 30 min and then added to the culture medium. The cells were washed at 2 h post infection and incubated with fresh medium. The cells were recovered after 24 h and visualized by microscopy. The scale bar indicates 250 μm. **k** The fluorescence intensities in **j** were quantitated as indicated. **l**–**n** Soluble human recombinant AXL, but not ACE2, blocks SARS-CoV-2 virus pseudotype infection in H1299 cells. **l** Human recombinant His-AXL was mixed with the GFP-labeled SARS-CoV-2 virus pseudotype for 30 min and then added to the culture medium of H1299 cells. The cells were washed at 2 h post infection and incubated with fresh medium. The cells were recovered after 24 h and visualized by microscopy. The scale bar indicates 250 μm. **m** The fluorescence intensities in **l** were quantitated as indicated. **n** Human recombinant HIS-AXL RP-His-AXL or HIS-ACE2 RP-HIS-ACE2 (25–200 µg/mL) was mixed with the SARS-CoV-2 virus pseudotype for 30 min and then added to the culture medium of H1299 cells. The cells were washed at 2 h post infection and incubated with fresh medium. The cells were recovered after 24 h, and viral RNA was examined by RT-qPCR. The data shown are representative results from three independent experiments (**a**–**n**, *n* = 3). The data are shown as the means ± SEM from three independent experiments. *P* values were calculated using two-way ANOVA (*P < 0.05).

To evaluate the significance of AXL in SARS-CoV-2 infection of pulmonary epithelial cells, we knocked down ACE2, AXL, LDLR, or EGFR (Supplementary information, Fig. S6) in H1299 cells by transfecting the cells with corresponding siRNAs and then infected the cells with the SARS-CoV-2 virus pseudotype (Fig. 5a, b). Downregulation of AXL, but not ACE2, LDLR or EGFR, drastically reduced SARS-CoV-2 virus pseudotype infection of H1299 cells at 24 h post infection (Fig. 5a, b), indicating that AXL is required for SARS-CoV-2 infection in these cells. Next, we established H1299 AXL-knockout (KO) or ACE2-KO cells using the CRISPR-Cas9 system (Fig. 5c). Knocking out AXL, but not ACE2, significantly blocked SARS-CoV-2 virus pseudotype infection in H1299 cells (Fig. 5d, e). Knocking out AXL reduced the SARS-CoV-2 virus pseudotype adsorption and internalization (Supplementary information, Fig. S5d, e), indicating that AXL is required for SARS-CoV-2 entry into pulmonary epithelial cells. Downregulation of AXL in ACE2-KO H1299 cells still significantly reduced SARS-CoV-2 virus pseudotype infection, indicating that AXL-mediated SARS-CoV-2 entry is ACE2-independent (Fig. 5f, g). To confirm that AXL directly induces viral infection independently of its ligand GAS6 or Protein S in serum,^30,33^ we performed control experiments and found that GAS6 and Protein S are dispensable in AXL-induced SARS-CoV-2 virus pseudotype infection. GAS6 does not bind SARS-CoV-2 (Supplementary information, Fig. S7a) or promote AXL-induced SARS-CoV-2 virus pseudotype infection (Supplementary information, Fig. S7b–e). Protein S in serum is also dispensable in AXL-induced SARS-CoV-2 virus pseudotype infection, as evaluated by using serum-free media (Supplementary information, Fig. S7f, g). Taken together, these results indicate that AXL specifically promotes SARS-CoV-2 entry into human pulmonary epithelial cells in an ACE2-independent manner.

Soluble human recombinant ACE2 has been found to reduce SARS-CoV-2 recovery from Vero cells, which express high levels of ACE2.^34^ To further confirm AXL’s indispensable role in SARS-CoV-2 infection of pulmonary and bronchial cells and determine the potential impact on clinical practice, we mixed soluble human recombinant ACE2 or AXL with the SARS-CoV-2 virus pseudotype and then infected HEK293T cells overexpressing ACE2 or AXL (Fig. 5h–k). Soluble human ACE2 and AXL blocked SARS-CoV-2 virus pseudotype infection in cells overexpressing ACE2 and AXL, respectively. However, soluble human ACE2 failed to block viral infection in cells overexpressing AXL, and vice versa (Fig. 5h–k), indicating that AXL’s function in mediating viral entry is likely independent of ACE2. Soluble human AXL, but not ACE2, blocked SARS-CoV-2 virus pseudotype infection in H1299 cells (Fig. 5l–n), confirming that AXL is required for SARS-CoV-2 entry into pulmonary epithelial cells.

### AXL is required for authentic SARS-CoV-2 entry into human cells

**Fig. 6:**
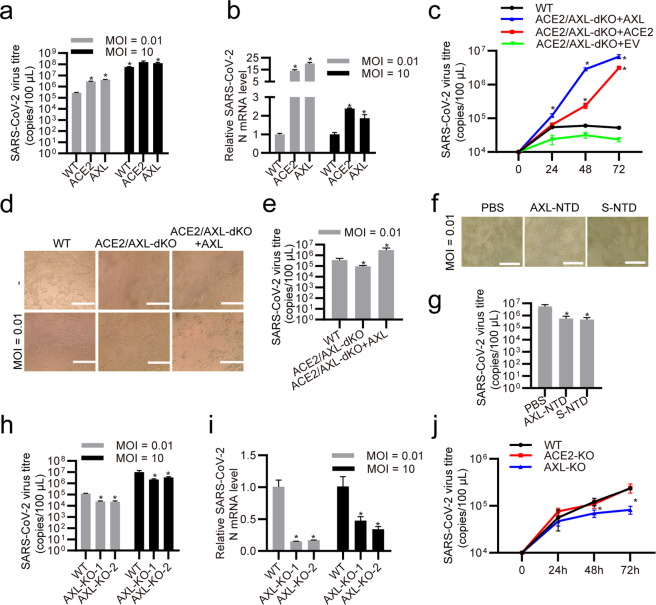
AXL promotes authentic SARS-CoV-2 infection in HEK293T and H1299 cells. **a**–**c** AXL promotes SARS-CoV-2 infection as potently as ACE2. **a** HEK293T cells stably expressing empty vector, ACE2 or AXL were infected with SARS-CoV-2 (MOI = 10 or 0.01), and SARS-CoV-2 progeny titers were measured in the cell supernatants by RT-qPCR assay at 72 h post infection with different concentrations of SARS-CoV-2. **b** Expression of the SARS-CoV-2 N gene was examined in the cells in **a** at 72 h post infection by RT-qPCR. **c** Control HEK293T cells (WT) and ACE2/AXL double-KO HEK293T cells stably expressing empty vector (ACE2/AXL-dKO+EV), ACE2 (ACE2/AXL-dKO+ACE2) or AXL (ACE2/AXL-dKO+AXL) were infected with SARS-CoV-2 (MOI = 0.01), and SARS-CoV-2 progeny titers were measured in the cell supernatants by RT-qPCR assay at 0, 24, 48 and 72 h post infection. **d**, **e** AXL promotes SARS-CoV-2 infection independent of ACE2. **d** Control HEK293T cells (WT), ACE2/AXL double-KO HEK293T cells (ACE2/AXL-dKO) and ACE2/AXL double-KO HEK293T cells stably expressing AXL (ACE2/AXL-dKO+AXL) were infected with SARS-CoV-2 (MOI = 0.01) and visualized by inverted microscopy at 72 h post infection. The scale bar indicates 250 μm. **e** SARS-CoV-2 progeny titers were measured in the supernatants of the cells in **d** by RT-qPCR assay at 72 h post infection. **f**, **g** Soluble human AXL-NTD and SARS-CoV-2 S-NTD block SARS-CoV-2 infection in HEK293T cells overexpressing AXL. **f** Human recombinant His-AXL-NTD or His-SARS-CoV-2 S-NTD was mixed with SARS-CoV-2 (MOI = 0.01) for 30 min and then added to the culture medium of HEK293T cells stably expressing AXL. The cells were visualized by inverted microscopy at 72 h post infection. The scale bar indicates 250 μm. **g** SARS-CoV-2 progeny titers were measured in the supernatants of the cells in **f** by RT-qPCR assay at 72 h post infection. **h**–**j** Knocking out AXL inhibits SARS-CoV-2 infection in H1299 cells. **h** Normal H1299 cells and two AXL-KO H1299 cell lines were infected with SARS-CoV-2 (MOI = 10 or 0.01), and SARS-CoV-2 progeny titers were measured in the cell supernatants by RT-qPCR assay at 72 h post infection with different concentrations of SARS-CoV-2. **i** Expression of the SARS-CoV-2 N gene was examined in the cells in (**h**) at 72 h post infection by RT-qPCR. **j** H1299, ACE2-KO or AXL-KO H1299 cells were infected with SARS-CoV-2 (MOI = 0.01), and SARS-CoV-2 progeny titers were measured in the cell supernatants by RT-qPCR assay at 0, 24, 48 and 72 h post infection. The data shown are representative results from three independent experiments (**a**–**j**, *n* = 3). The data are shown as the means ± SEM from three independent experiments. *P* values were calculated using two-way ANOVA (*P < 0.05).

Intact SARS-CoV-2 virions may act differently than the SARS-CoV-2 virus pseudotype.^12,35^ To confirm AXL’s role in mediating the infection of authentic SARS-CoV-2 virus, HEK293T cells stably expressing human ACE2, human AXL, murine AXL, rhesus macaque AXL or an empty vector were infected with authentic SARS-CoV-2 virus (Fig. 6a–c, Supplementary information, Fig. S3g). Viral infection was significantly enhanced in HEK293T cells overexpressing human ACE2 or human AXL, but not in HEK293T cells expressing the empty vector (Fig. 6a–c) or murine or rhesus macaque AXL (Supplementary information, Fig. S3h), reproducing the results observed using the SARS-CoV-2 virus pseudotype and confirming that both ACE2 and AXL are SARS-CoV-2 receptors. To test whether AXL promotes SARS-CoV-2 virus infection in an ACE2-dependent manner, we first double knocked out ACE2 and AXL in HEK293T cells, which express low levels of both proteins, and then infected the cells with SARS-CoV-2 virus (Fig. 6d). Viral infection was slightly decreased by ACE2/AXL double-KO in HEK293T cells (Fig. 6d, e). Overexpressing AXL in the double-KO HEK293T cells greatly promoted viral infection (Fig. 6d, e), which reached a level similar to that in HEK293T cells overexpressing AXL (Fig. 6a, e), indicating that ACE2 is dispensable in AXL-promoted SARS-CoV-2 virus infection. Further evidence acquired by measuring the viral RNA of adsorbed viruses to cells (Supplementary information, Fig. S5f) and of internalized viruses in cells (Supplementary information, Fig. S5g) indicates that AXL enhance authentic SARS-CoV-2 infection by promoting its attachment and entry to the host cells. To confirm that the binding between AXL and SARS-CoV-2 S plays an indispensable role in authentic SARS-CoV-2 infection and to determine the potential impact on clinical practice, we mixed soluble human recombinant AXL NTD or recombinant SARS-CoV-2 S NTD with SARS-CoV-2 virus and then infected HEK293T cells overexpressing AXL (Fig. 6f, g). Both recombinant proteins reduced SARS-CoV-2 infection in HEK293T cells overexpressing AXL, confirming that binding between AXL and SARS-CoV-2 S through their NTDs is essential for SARS-CoV-2 entry into cells (Fig. 6f, g).

To evaluate the significance of AXL in SARS-CoV-2 infection of pulmonary epithelial cells, we infected AXL-KO H1299 cells with authentic SARS-CoV-2 virus (Fig. 6h–j; Supplementary information, Fig. S5h, i). AXL KO reduced the SARS-CoV-2 adsorption and internalization (Supplementary information, Fig. S5h, i), indicating that AXL is required for SARS-CoV-2 entry into pulmonary epithelial cells. Knocking out of AXL, but not ACE2, greatly reduced viral infection in H1299 cells (Fig. 6h–j). Taken together, these results indicate that AXL is required for SARS-CoV-2 infection in pulmonary epithelial cells.

### AXL promotes authentic SARS-CoV-2 infection in primary lung epithelium and in COVID-19 patients

**Fig. 7:**
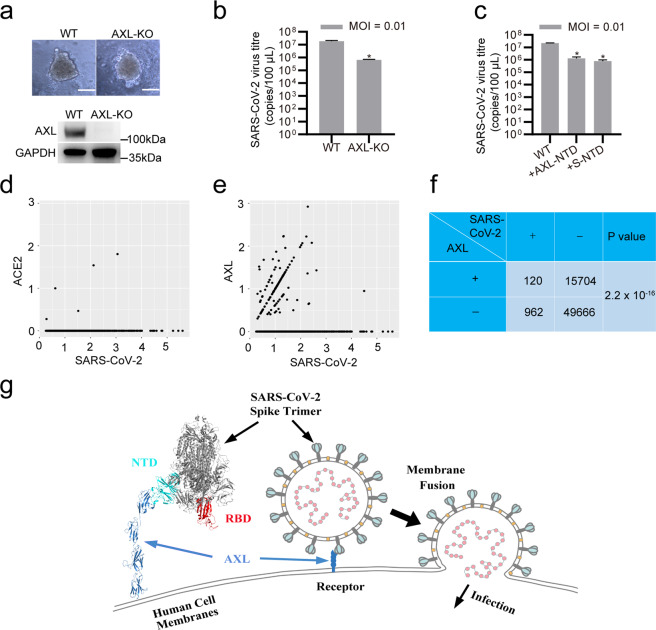
AXL promotes SARS-CoV-2 infection in primary lung epithelium and in COVID-19 patients. **a**–**c** Knocking out AXL inhibits SARS-CoV-2 infection in human primary lung epithelial organoids. **a** Control and AXL-KO human primary lung cells were established using the CRISPR-Cas9 system, allowed to form spheroids, and visualized by inverted microscopy (upper panel). The knockdown efficiencies were evaluated using western blotting assay with an antibody against ACE2 or AXL (lower panel). The scale bar indicates 300 μm. **b** The spheroids in **a** were infected with SARS-CoV-2, and SARS-CoV-2 progeny titers were measured in the primary lung organoid supernatants by RT-qPCR at 72 h post infection. **c** Human recombinant His-AXL-NTD or His-SARS-CoV-2 S-NTD was mixed with SARS-CoV-2 for 30 min and then added to the culture medium of primary lung organoids. SARS-CoV-2 progeny titers were measured in the primary lung organoid supernatants by RT-qPCR at 72 h post infection. **d**–**f** AXL expression is associated with SARS-CoV-2 infection in COVID-19 patients. The correlation between **d** ACE2 or **e** AXL and SARS-CoV-2 expression in SARS-CoV-2-positive cells from COVID-19 patients was evaluated at the single-cell level. **f** The total counts of SARS-CoV-2-positive, AXL-positive and SARS-CoV-2-negative cells in **e** are listed. The χ² test was used to evaluate the significance of co-expression. **g** Schematic of AXL-mediated SARS-CoV-2 receptor recognition and/or membrane fusion. The data shown are representative results from three independent experiments (**a**–**c**, *n* = 3). The data are shown as the means ± SEM from three independent experiments. *P* values were calculated using two-way ANOVA for comparisons among multiple groups or *t*-tests for comparisons between two groups (*P < 0.05).

To test whether AXL promotes SARS-CoV-2 infection in primary lung epithelium, we established human primary lung epithelial spheroids using epithelial cells isolated from para-carcinoma lung tissues obtained from lung cancer patients (Fig. 7a). Knocking out AXL in the primary cells that formed spheroids (Fig. 7a) greatly reduced authentic SARS-CoV-2 virus infection (Fig. 7b), indicating that AXL is required for SARS-CoV-2 entry into primary pulmonary epithelium. Moreover, the recombinant AXL NTD and the recombinant SARS-CoV-2 S NTD protein each significantly decreased SARS-CoV-2 virus infection in human primary lung epithelial spheroids (Fig. 7c).

To establish the correlation between AXL expression and SARS-CoV-2 infection in COVID-19 patients, we reanalyzed the expression levels of AXL in a single-cell mRNA sequencing dataset of bronchoalveolar lavage fluid (BALF) cells from COVID-19 patients^36^ (Fig. 7d–f). Both ACE2 (Fig. 7d) and AXL (Fig. 7e) expression levels were well correlated with SARS-CoV-2 levels in infected BALF cells. However, there were significantly more AXL-positive cells (120/1082 cells) than ACE2-positive cells (5/1082 cells) among SARS-CoV-2-positive BALF cells (Fig. 7d, e). The χ^2^ test showed that AXL-positive cells were more vulnerable than AXL-negative cells to SARS-CoV-2 infection (*P* = 2.2 × 10^−16^) in COVID-19 patients (Fig. 7f).

Taken together, our results not only confirm that the human tyrosine-protein kinase receptor AXL specifically interacts with the SARS-CoV-2 S glycoprotein but also clarify its indispensable role in facilitating SARS-CoV-2 entry into human pulmonary epithelial cells in an ACE2-independent manner (Fig. 7g).

## Discussion

COVID-19 primarily causes respiratory system illness; however, an increasing number of case reports on SARS-CoV-2 infection have shown that it can affect almost all of the body’s primary organs, including the lungs, pharynx, heart, liver, brain, and kidneys.^7^ SARS-CoV-2 particles were originally visualized in ultrathin sections of human airway epithelial cells.^1^ Later, SARS-CoV-2 RNA was detected in esophageal, stomach, duodenal, and rectal specimens^8^ as well as in kidneys.^7^ These results are consistent with our reanalyzed single-cell sequencing data showing that ACE2 is highly expressed in the kidneys and digestive system. However, ACE2 expression was expressed in fewer than 1% of cells in other tissues, including the heart (0.3%), liver (0.1%), brain (0%), lungs (0.1%) and trachea (0.2%). Accordingly, we hypothesized that additional proteins may exist that are responsible for viral entry in these tissues.

In the current study, we found that SARS-CoV-2 is able to utilize either ACE2 or AXL for entry into human cells. AXL belongs to the family of TAM phosphatidylserine receptors. Similar to TIM-family phosphatidylserine receptors,^37^ AXL has been reported to increase the transduction efficiencies of lentiviral vectors pseudotyped with several types of envelope proteins using an “apoptotic mimicry” machinery,^38^ possibly utilizing the ligand Gas6 or Protein S in serum to bridge its interaction with phosphatidylserine-modified viral envelope proteins.^33^ However, many other viruses and virus pseudotypes, including vesicular stomatitis virus, Lassa virus, herpes simplex virus 1, influenza A virus, Oliveros virus, and SARS-CoV, do not use this general machinery to enter host cells.^33,37,39^ Importantly, overexpression of TIM1 does not enhance SARS-CoV infection.^37^ Given this information and our data indicating that other TIM/TAM family members, GAS6 and Protein S in serum all fail to induce SARS-CoV-2 virus pseudotype infection, it is very unlikely that SARS-CoV-2 utilizes this apoptotic mimicry mechanism to infect cells. Taken together, the findings show that AXL specifically promotes SARS-CoV-2 entry into human pulmonary epithelial cells in a mechanism largely independent of virion-associated phosphatidylserine.

AXL is widely expressed in almost all human organs. In particular, in human pulmonary and bronchial epithelial tissue and cells, AXL expression is much higher than ACE2 expression. Given that AXL was not co-expressed with ACE2 or TMPRSS2 in the human lungs or trachea, that downregulation of AXL in ACE2-KO H1299 cells significantly reduced SARS-CoV-2 virus pseudotype infection, and that neither AXL nor ACE2 could block viral infection when the other protein was overexpressed, AXL’s function in mediating SARS-CoV-2 infection is very likely independent of ACE2. This probable scenario was further confirmed through authentic SARS-CoV-2 infection assays. Nevertheless, further investigation is needed to determine whether AXL and ACE2 utilize the same co-factors or are involved in similar infection processes.

Since the binding, internalization and infection of SARS-CoV-2 was greatly reduced but not completely abolished in AXL-KO H1299 cells, additional receptor(s) other than AXL and ACE2 which mediates viral entry may exist. Several proteins have been recently identified to interact with SARS-CoV-2 S, including lectin receptors and multiple innate immune receptors,^40,41^ heparan sulfate,^42,43^ neuropilins,^44,45^ asialoglycoprotein receptor 1 and Kremen protein 1.^46^ However, most of them lack virology-related evidence to support their roles as SARS-CoV-2 entry factors. Further investigations are needed to establish their roles in mediating SARS-CoV-2 viral entry. Among them, neuropilin-1 actually functions as a co-factor rather than a receptor to facilitate ACE2- and TMPRSS2-mediated SARS-CoV-2 infection.^44,45^ Of the reported candidate receptors, we recovered only trace amounts of heparan sulfate and did not recover the others in our TAP-MS analysis of the SARS-CoV-2 S-associated protein complex, indicating that AXL is a major receptor for SARS-CoV-2 infection.

Several genome-wide CRISPR screens have been recently performed to identify host factors required for SARS-CoV-2 infection in Vero-E6,^47^ Huh7.5.1,^48^ and A549-ACE2 cells.^48,49^ Since all the screens have been performed in cells known to express high levels of ACE2, they have been unlikely to identify novel receptors that mediate SARS-CoV-2 entry in an ACE2-independent manner. None of the above-mentioned candidate receptors has been identified as one of the top candidates in these screens. Future screens performed in cells vulnerable to SARS-CoV-2 infection and expressing low levels of ACE2 (e.g., H1299 cells) may contribute to the identification of novel receptors.

Since the outbreak of COVID-19, extensive effort has been devoted to developing drugs that target the human cell virus receptor ACE2. Consistent with the low expression levels of ACE2 in human pulmonary and bronchial epithelial cells, downregulation of ACE2 elicited a minor effect with regard to reducing SARS-CoV-2 virus pseudotype infection of H1299 cells, indicating that ACE2 might not be a good drug target for the lungs and bronchi. However, since ACE2 is highly expressed in the jejunum, duodenum and kidneys, combined therapy targeting ACE2 and AXL might achieve good antiviral activity in these organs. As AXL is overexpressed in numerous cancer types, it has already been pursued as a drug target. However, we note that most reported AXL inhibitors target the AXL kinase domain and thus should not have efficacy against SARS-CoV-2 infection because the virus interacts with the extracellular Ig-like domains of AXL. Alternative approaches include targeting the AXL extracellular Ig-like domains and the region of the SARS-CoV-2 S protein that interacts with AXL. Clinical-grade human recombinant soluble AXL may be useful for blockade of viral infection. Based on our data, AXL is likely to bind the NTD of SARS-CoV-2 S, while ACE2 binds to the RBD. Interestingly, a recent study identified a potent neutralizing human antibody that binds to the NTD of SARS-CoV-2 S.^19^ Moreover, another recent study showed that of 84 monoclonal antibodies isolated from three COVID-19 patients, 33 bound strongly to SARS-CoV-2 S but did not bind the RBD.^18^ Considering that the NTD and RBD account for major portions of SARS-CoV-2 S S1, these antibodies likely bind to the NTD of SARS-CoV-2 S. These reports highlight the importance of the NTD of SARS-CoV-2 S during viral infection and may support an important role of AXL during infection of human pulmonary and bronchial tissues.

Taken together, our findings demonstrate AXL’s indispensable role in facilitating SARS-CoV-2 infection. We anticipate that upon further validation and mechanistic elucidation, our findings will contribute to the development of therapeutic solutions for COVID-19.

## Materials and methods

### Cell culture, plasmid construction, transfection

HEK293T, NCI-H1299, BEAS-2B, NCI-H292, CALU-3, HepG2, HGC-27, MDA-MB-231 and SH-SY5Y cells were purchased from the American Type Culture Collection (ATCC, USA). NCI-H460 and HL7702 cells were purchased from the Cell Bank of the Chinese Academy of Sciences, China. HEK293T, HepG2 and MDA-MB-231 cells were maintained in Dulbecco’s modified Eagle’s medium (DMEM); NCI-H1299, NCI-H292, NCI-H460, HL7702 and HGC-27 cells were maintained in RPMI-1640 medium; BEAS-2B cells were maintained in Bronchial Epithelial Cell Growth Basal Medium (Lonza, Switzerland); CALU-3 cells were maintained in Eagle’s Minimum Essential Medium (ATCC, USA); and SH-SY5Y cells were maintained in Eagle’s Minimum Essential and F12 Medium (50:50) (ATCC, USA). All culture media contained 10% fetal bovine serum (FBS, Gibco, Australia) and were supplemented with 1% penicillin and streptomycin (Sigma-Aldrich, UK).

The pDEST-SFB-SARS-CoV-2-S and pCMV-MYC-ACE2, pCMV-MYC-AXL, pCMV-MYC-EGFR and pCMV-MYC-LDLR plasmids were synthesized directly. The influenza A virus HA plasmid, a generous gift from Dr. Qiyun Zhu (Lanzhou Veterinary Research Institute, Chinese Academy of Agricultural Sciences), was cloned into the pDEST-SFB vector. Deleted and truncated SARS-CoV-2-S and AXL constructs were generated by PCR-based amplification using the wild-type construct as the template. All constructs were confirmed by sequencing. The plasmids were transiently transfected into the indicated cells using jetPRIME DNA transfection reagents (Polyplus, France) according to the manufacturer’s instructions.

siRNAs against ACE2 (si-ACE2-1: 5ʹ-GCGAGUGGCUAAUUUGAAAtt-3ʹ, si-ACE2-2: 5ʹ-GCACUUUGUCAAGCAGCUAtt-3ʹ), AXL (si-AXL-1: 5ʹ-GGGUGGAGGUUAUCCUGAAtt-3ʹ, si-AXL-2: 5ʹ- CCUGUGGUCAUCUUACCUUtt-3ʹ), EGFR (si-EGFR-1: 5ʹ-GUCCGCAAGUAAGAAGUtt-3ʹ, si-EGFR-2: 5ʹ-GCAACAUGUCGAUGGACUUtt-3ʹ), and LDLR (si-LDLR-1: 5ʹ-CGGCUUAAGAACAUCAACAtt-3ʹ, si-LDLR-2: 5ʹ-GGGUCUUCCUUCUAUGGAAtt-3ʹ) were synthesized. Plasmid transfection and siRNA transfection were performed. Briefly, cells were cultured in six-well plates, and 2 μL of plasmid (1 μg/μL) or siRNA (50 nM) was mixed with 200 μL of jetPRIME buffer for 10 s. 5 μL of jetPRIME transfection reagent (Polyplus, France) was added, and the cells were incubated for 10 min. The mixtures were then added to the wells of six-well plates and incubated for 24 h.

sgRNAs against ACE2 (sg-ACE2: 5ʹ-GAAAGCTGGAGATCTGAGGT-3ʹ) and AXL (sg-AXL-1: 5ʹ- GCGAAGCCCATAACGCCAAGG-3ʹ, sg-AXL-2: 5ʹ-GGTTCAGGGAGAGCCCCCCG-3ʹ) were synthesized and cloned into the pLenti-V2 vector. The lentivirus was packaged in HEK293T cells, condensed by ultra-centrifugation and used to infect H1299 cells. The cells were selected with puromycin (2 μg/mL) for 2 days and subcloned to form single colonies. KO cell clones were screened by western blotting assay to verify the loss of ACE2 or AXL expression.

### Tandem affinity purification

H1299, BEAS-2B and HEK293T cells stably expressing an N-terminal SFB-fused SARS-CoV-2 S protein or control influenza A virus HA were selected via culture in medium containing 2 μg/mL puromycin. Protein expression was confirmed by immunostaining and western blotting assay as described previously.^50^ For TAP, the membrane-bound and soluble proteins of 1 × 10^8^ cells were extracted using a membrane/soluble protein isolation kit (Beyotime, China) with protease inhibitors at 4 °C. The lysates were combined and incubated with streptavidin-conjugated beads (Thermo Fisher Scientific, USA) for 2 h at 4 °C. The beads were washed three times with 1× NETN buffer (20 mM Tris-HCl, pH 8.0, 100 mM NaCl, 1 mM EDTA, 0.5% Nonidet P-40), and the bound proteins were eluted with NETN buffer containing 2 mg/mL biotin (Sigma-Aldrich, USA) for 2 h at 4 °C. The eluates were incubated with S-protein beads (Millipore, USA) for 1 h; the beads were washed three times with NETN buffer and subjected to sodium dodecyl sulfate-polyacrylamide gel electrophoresis. Each pull-down sample was electrophoresed. Each whole band was excised as one sample and subjected to in-gel trypsin digestion and liquid chromatography (LC)-MS.

### LC and MS

LC and MS were performed as described previously.^21,22^ Briefly, gel bands excised as described above were cut into ~1-mm^3^ pieces, which were then subjected to in-gel trypsin digestion^51^ and dried. The samples were reconstituted in 5 μL of high-performance LC solvent A (2.5% acetonitrile and 0.1% formic acid). A nanoscale reverse-phase high-performance LC capillary column was created by packing 5-μm C18 spherical silica beads into a fused silica capillary (100 μm inner diameter × ~20 cm length) using a flame-drawn tip. After the column was equilibrated, each sample was loaded using an autosampler. A gradient was formed, and peptides were eluted with increasing concentrations of solvent B (97.5% acetonitrile and 0.1% formic acid); the peptides were subjected to MS as they eluted.

For MS with an Orbitrap Fusion Lumos, the source was operated at 1.9 kV, with no sheath gas flow and with the ion transfer tube at 350 °C. The data-dependent acquisition mode was used. The survey scan was conducted from m/z 350 to 1500, with a resolution of 60,000 at m/z 200. The 20 most intense peaks with charge states of 2 and greater were acquired with collision-induced dissociation with a normalized collision energy of 30% and one microscan; the intensity threshold was set at 1000. MS2 spectra were acquired with a resolution of 15,000. The peptides were detected, isolated, and fragmented to produce a tandem mass spectrum of specific fragment ions for each peptide.

### MS data analysis

The MS peptide sequences and hence the protein identities were determined by matching fragmentation patterns in protein databases using the Mascot software program (Matrix Science, USA). The enzyme specificity was set to partially tryptic with two missed cleavages. The peptide modifications included carboxyamidomethylation (cysteines, variable), oxidation (methionine, variable), phosphorylation (S, T, Y, H, variable) and acetylation (N-term, K, variable). The mass tolerance was set to 20 ppm for precursor ions and fragment ions. UniProt was the database searched (*Homo sapiens*, SARS-CoV-2 and influenza A virus (A/Guangzhou/39715/2014(H5N6))). Spectral matches were filtered for a false discovery rate of less than 1% at the peptide level using the target-decoy method,^52^ and protein inference was considered following the general rules,^53^ with manual annotation applied when necessary. This same principle was used for protein isoforms when they were present; in general, the longest isoform is reported. Interaction filtration was further performed using the MUSE algorithm as described previously^21,22^ to assign quality scores for the identified protein-protein interactions (PPIs). TAP-MS performed under identical experimental conditions for influenza A virus HA from H1299 cells was used as a true negative control for the MUSE analysis.

### Analysis of protein expression levels in human tissue cells

ACE2 and AXL expression in cells from human tissues was analyzed using a recently published single-cell mRNA sequencing dataset consisting of 232,905 single cells from all major adult organs after the batch gene background was removed and after cells with fewer than 500 detected transcripts were filtered out.^15^ The matrices for the digital gene expression data were ln(CPM/100 + 1)-transformed, and downstream procedures for filtering and dimensional reduction were performed using Seurat v3.1.0.^54^ All the genes were used for initial principal component analysis, and the top 10 principal components were used for nonlinear dimensional reduction (*t*-distributed stochastic neighbor embedding, *t*SNE) analysis.

### Computational modeling of protein–protein complexes

Protein-protein docking calculations were performed using HADDOCK.^23^ For each receptor, 600,000 docking poses were randomly generated and rigid-body minimized in the first stage, after which the 400 poses with the lowest HADDOCK scores were selected for optimization of the protein-protein interface with simulated annealing. After further refinement of these poses in an explicit 8-Å water layer environment, clustering analysis was conducted, and the lowest-scoring poses for each of the top 20 largest clusters were selected. One additional filtering step was performed by building atomistic models of the interactions of the full-length receptors with SARS-CoV-2 S and excluding any conformations leading to potential steric clashes.

In the next stage, MD simulations were carried out to assess the stability of these docking poses. The simulation systems were established using CHARMM,^55^ and the selected complexes were solvated in a periodic 210 × 210 × 10-Å^3^ cubic TIP3P water box and neutralized with extra K^+^ or Cl^−^ ions. The systems were subjected to 11-ns NPT simulations with the CHARMM36m protein force field^56^ using OpenMM.^57^ Non-bonded interactions were truncated at 12 Å with smooth switching from 10 Å, and electrostatic interactions were calculated using the particle mesh Ewald (PME) method. The MD trajectories evolved at 300 K with a 2-fs timestep in which bonds involving H atoms were constrained. To provide a rough estimation of the binding affinity between the candidate receptors and full-length SARS-CoV-2 S, MM/PBSA calculations were carried out using the last 1-ns MD trajectories, as follows:

$$S = \langle {\Delta}{\mathrm{MM}}\rangle + \langle {\Delta}G_{{\mathrm{PB}}}\rangle + {\upgamma}\langle {\Delta}{\mathrm{ASA}}\rangle ,$$

where ΔMM, Δ*G*_PB_, and ΔASA are the differences in non-bonded interaction energy, solvation free energy based on Poisson-Boltzmann (PB) calculations, and solvent-accessible surface area as the protein-protein complexes form, respectively; γ equals 0.00572 kcal/mol/Å^2^; and the brackets indicate the ensemble average.

### Western blotting, immunoprecipitation and immunofluorescence

Cells were harvested and lysed in NETN buffer on ice for 30 min. After measuring the protein concentration using a BCA kit (Thermo Fisher Scientific, USA), 5× loading buffer (Beyotime, China) was added, and the mixture was boiled for 15 min. After 10% sodium dodecyl sulfate-polyacrylamide gel electrophoresis, the proteins were transferred onto PVDF membranes (Millipore, USA). The membranes were incubated with the indicated primary antibodies at 4 °C overnight, washed three times with PBS-T buffer (1× PBS, 0.05% V/V TWEEN 20) for a total of 15 min and incubated with secondary antibodies (1:2000, Cell Signaling Technology, CST, USA) for 1 h at room temperature. The signals were detected using an enhanced chemiluminescence (ECL) kit (Pierce, USA).

For immunoprecipitation (IP) and co-IP assays, 1 × 10^7^ cells were lysed in NETN buffer on ice for 30 min. The cell lysates were precleared by incubation with 5 μL of magnetic beads (Millipore, USA) for 1 h and were then incubated with the indicated antibodies at 4 °C with rotation overnight. After centrifugation, the supernatant was incubated with 10 μL of magnetic beads at 4 °C with rotation for 1 h. The beads were washed three times with cold NETN buffer using a magnetic separator (Millipore, USA), and elution was then performed with 40 μL of protein lysis buffer (Beyotime, China). Ten microlitres of 5× loading buffer (Beyotime, China) was added, and the mixture was boiled for 15 min and subjected to western blotting assay.

For immunofluorescence assays, cells were seeded in a cell culture dish, fixed and permeabilized at 4 °C for 30 min. After incubation with the indicated antibodies at 4 °C overnight, the cells were washed with PBS twice, stained with goat-anti-rabbit Fluorescein isothiocyanate-labeled IgG or goat-anti-mouse rhodamine-labeled IgG (1:200, Proteintech, China) at 4 °C for 2 h, and subjected to 4′6-diamidino-2-phenylindole (DAPI) staining (Sigma-Aldrich, USA). The cells were viewed using an Olympus IX73 Microscope Imaging System (Olympus, Japan).

The following primary antibodies were used: anti-MYC (1:2000, CST, #2276, USA), anti-FLAG (1:2000, CST, #14793, USA), anti-His (1:2000, Abcam, ab18184, USA), anti-AXL (1:1000, Abcam, ab219651, USA), anti-LDLR (1:1000, Abcam, ab52818, USA), anti-ACE2 (1:1000, Abcam, ab15348, USA), anti-EGFR (1:1000, Abcam, ab52894, USA), anti-GAPDH (1:2000, Abcam, ab181602, USA), anti-SARS-CoV-2 S (1:100, Abcam, ab272504), anti-CAV1 (1:500, CST, #3267, USA), anti-EEA1 (1:2000, CST, C45B10, USA), anti-APPL1 (1:2000, CST, D83H4, USA), anti-CLTC (1:2000, CST, D3C6, USA) and anti-STX6 (1:2000, CST, C34B2, USA).

### Biolayer interferometry quantification assay

The binding between the SARS-CoV-2 S NTD and AXL was measured by BLI using Octet RED96E systems (ForteBio, USA). All the samples were diluted with working buffer, a detergent-based kinetic buffer (PBS + 0.02% Tween 20, 0.1% BSA, 0.05% sodium azide). Anti-His (HIS1K) biosensors (18‐5120, ForteBio, USA) were hydrated in working buffer. All BLI experiments were set up with the load sample fixed to the anti-His biosensor surface (His-tagged SARS-CoV-2 NTD) and the analyte sample (FLAG-AXL) in solution. The concentration range of the load sample was 10–50 µg/mL (~µM range), and the concentration range of the analyte sample was 0.01–100 × KD. FLAG-AXL samples were then associated with the biosensors, and association and dissociation profiles were obtained. Data were acquired (in kinetics mode) and analyzed using data acquisition software v12.0 (ForteBio, USA). Binding was calculated from the response amplitude (wavelength shift in nm) obtained in the first 100 s of each step.

### SARS-CoV-2 virus pseudotype production and infection

A SARS-CoV-2 virus pseudotype was packaged and used to infect cells as previously described.^58^ Briefly, HEK293T cells were cultured in 10-cm plates pre-coated with poly-L-lysine and incubated with DMEM supplemented with 10% FBS, penicillin/streptomycin and L-glutamine. The next day, the cells were co-transfected with psPAX2, pLenti-GFP, and SARS-CoV-2 S plasmids using jetPRIME DNA transfection reagents (Polyplus, France) according to the manufacturer’s instructions. The supernatants were collected at 48 and 72 h post transfection, mixed with PEG overnight, passed through a 0.45-μm filter, centrifuged at 500× *g* for 5 min, aliquoted and stored at –80 °C.

To transduce cells with the SARS-CoV-2 virus pseudotype, HEK293T or H1299 cells were seeded into 24-well plates, transfected with the indicated plasmids or siRNAs overnight, and then infected with the SARS-CoV-2 virus pseudotype for 24 h. The cells were washed 3 times and viewed using an Olympus IX73 Microscope Imaging System (Olympus, Japan). GFP-positive cells were considered to be infected by the SARS-CoV-2 virus pseudotype.^58^ To titrate the SARS-CoV-2 virus pseudotype after infection, H1299 cells were cultured in 24-well plates. Human recombinant HIS-AXL (25–200 µg/mL, amino acids 1–449, RP-HIS-AXL) or HIS-ACE2 (RP-HIS-ACE2) was mixed with the SARS-CoV-2 virus pseudotype (10^7^ pfu, Multiplicity of Infection, MOI = 5) for 30 min, and the mixture was then added to the culture medium of H1299 cells. The cells were washed at 2 h post infection and incubated with fresh medium. The cells were recovered after 24 h, and viral RNA was assayed by quantitative real-time PCR (RT-qPCR).

### Authentic SARS-CoV-2 infection of human cells

Authentic SARS-CoV-2 was isolated from a patient in Shanghai with COVID-19. We plaque-purified and massively expanded the initial generation in Vero-E6 cells and stored the virus at –80 °C. We deep-sequenced the strain and named it SARS-CoV-2/SH01/human/2020/CHN (GenBank accession number: MT121215). Compared with the Wuhan strain, this strain has the same gene sequence encoding the S glycoprotein but has one mutation in each of the following genes: non-structural protein (nsp) 3, nsp8 and nsp14. All authentic SARS-CoV-2 infection assays were performed using these early passages of SARS-CoV-2 to ensure the consistency of our experiments.

HEK293T and H1299 cells were seeded in 96-well plates (1 × 10^4^ cells per well) in DMEM containing 2% FBS. The cells were infected with 1 × 10^6^ pfu/mL or 1 × 10^3^ pfu/mL SARS-CoV-2 (MOI = 10 or 0.01, respectively) at 37 °C for 1 h, washed with 1× PBS three times and cultured in complete medium for 72 h. Cytopathic effects were viewed using an EVOS M5000 microscope (Thermo Fisher Scientific, USA), and the supernatant was collected and lysed using TRIzol LS (Thermo Fisher Scientific, USA) before analysis by RT-qPCR for SARS-CoV-2 N RNA detection. The primers were as follows: SARS-CoV-2-N-F, GGGGAACTTCTCCTGCTAGAAT; SARS-CoV-2-N-R, CAGACATTTTGCTCTCAAGCTG; and SARS-CoV-2-N-probe, 5′-FAM- TTGCTGCTGCTTGACAGATT-TAMRA-3′.

All the experiments were performed in BSL-3 labs.

### Evaluation of SARS-CoV-2 adsorption and internalization

The kinetics of the viral entry were studied by quantifying SARS-CoV-2 virus pseudotype or authentic SARS-CoV-2 binding and internalization as previously described.^59,60^ ACE2/AXL double-KO HEK293T cells, ACE2/AXL double-KO HEK293T cells stably expressing AXL, control H1299 cells and H1299-AXL-KO cells were infected with SARS-CoV-2 virus pseudotype or authentic SARS-CoV-2 (MOI = 10) at 4 °C for 2 h and then extensively washed with chilled PBS to remove unattached viral particles. For binding assays, the washed cells were lysed to determine the amount of virus attached. For internalization assays, the washed cells were placed back to 37 °C for 2 h to allow internalization of attached virus. Then the cells were extensively washed with chilled PBS, followed by 0.05% trypsin-EDTA and stripping buffer (0.2% glacial acetic acid and 500 mM NaCl) to remove surface-bound virus. The cells were then lysed to assess viral internalization by determining the number of viral copies within the cells. The expression of GFP or SARS-CoV-2 N gene for SARS-CoV-2 virus pseudotype or authentic SARS-CoV-2, respectively, were normalized to the expression of the GAPDH to assess the viral adsorption and internalization.

### Primary lung tissue spheroid formation

Human lung cancer tissues were obtained from a patient in the First Affiliated Hospital of the Zhejiang University School of Medicine. Para-carcinoma tissues were digested and filtered through a 40-μm cell strainer (Corning, USA) to obtain single-cell suspensions. Primary lung cells were cultured in Alveolar Epithelial Cell Medium (ScienCell, USA). The primary lung cells were infected with lentivirus-packaged control sgRNA or AXL-targeting sgRNA and screened to obtain AXL-KO cells. Cells were seeded at a density of 1000 cells/well in 100 μL into 96-well spheroid microplates (Corning, USA) and incubated in a humidified incubator at 37 °C under 5% CO_2_ for the formation of a single spheroid per well. Ninety-six hours after cell seeding, primary lung cell spheroids had formed.

### Quantitative real-time PCR

Cells were harvested, and total RNA was extracted using TRIzol Reagent (Invitrogen, USA). RNA from each sample was reverse-transcribed into cDNA using a PrimeScript RT Master Mix Kit (Takara, Japan). RT-qPCR was performed using a Q5 real-time PCR system (Applied Biosystems, USA) with SYBR Green Master Mix (Toyobo, Japan). The data were normalized to the GAPDH expression level in each sample. The sequences of the primers synthesized for RT-qPCR were as follows: ACE2: F: CGAAGCCGAAGACCTGTTCTA, R: GGGCAAGTGTGGACTGTTCC; AXL: F: GTGGGCAACCCAGGGAATATC, R: GTACTGTCCCGTGTCGGAAAG; LDLR: F: TCTGCAACxATGxGCTAGAGACT, R: TCCAAGCATTCGTTGGTCCC; EGFR: F: AGGCACGAGTAACAAGCTCAC, R: ATGAGGACATAACCAGCCACC; and GAPDH: F: GGAGCGAGATCCCTCCAAAAT, R: GGCTGTTGTCATACTTCTCATGG.

### Statistical analysis and ethics statement

No pre-processing of data was performed. All the western blotting, immunofluorescence and RT-qPCR data were obtained from at least three repeated experiments. The data were analyzed using Prism 5.0 software (GraphPad, USA) and are presented as the means ± SEM. Statistical significance between two groups was determined by unpaired two-tailed Student’s *t*-test. Multiple-group comparisons were performed using one-way analysis of variance (ANOVA). Differences were considered to be significant for *P* < 0.05 (indicated with an asterisk (*)). This study was approved by the Ethics Committee of Westlake University.

## Supplementary information

Supplementary information, Fig. S1

Supplementary information, Fig. S2

Supplementary information, Fig. S3

Supplementary information, Fig. S4

Supplementary information, Fig. S5

Supplementary information, Fig. S6

Supplementary information, Fig. S7

Supplementary information, Table Legends

Supplementary information, Table S1

Supplementary information, Table S2

Supplementary information, Table S3

Supplementary information, Table S4

## Data Availability

The MS proteomics data have been deposited in the ProteomeXchange Consortium (http://proteomecentral.proteomexchange.org) via the PRIDE partner repository (project name: LC-MS/MS proteomic analysis of SARS-CoV-2 Spike glycoprotein, accession number: PXD018908). The source data for all figures and tables will be made available upon publication. All other data supporting the findings of this study are available from the corresponding authors on reasonable request.

## References

[1] Zhou PYX (2020). A pneumonia outbreak associated with a new coronavirus of probable bat origin. Nature.

[2] Zhu N (2020). A novel coronavirus from patients with pneumonia in China, 2019. N. Engl. J. Med..

[3] Ksiazek TG (2003). A novel coronavirus associated with severe acute respiratory syndrome. N. Engl. J. Med..

[4] Zaki AM, van Boheemen S, Bestebroer TM, Osterhaus AD, Fouchier RA (2012). Isolation of a novel coronavirus from a man with pneumonia in Saudi Arabia. N. Engl. J. Med..

[5] Zou L (2020). SARS-CoV-2 viral load in upper respiratory specimens of infected patients. N. Engl. J. Med..

[6] Lamers MM (2020). SARS-CoV-2 productively infects human gut enterocytes. Science.

[7] Puelles VG (2020). Multiorgan and renal tropism of SARS-CoV-2. N. Engl. J. Med.

[8] Lin L (2020). Gastrointestinal symptoms of 95 cases with SARS-CoV-2 infection. Gut.

[9] Li F (2016). Structure, function, and evolution of coronavirus spike proteins. Annu. Rev. Virol..

[10] Belouzard S, Chu VC, Whittaker GR (2009). Activation of the SARS coronavirus spike protein via sequential proteolytic cleavage at two distinct sites. Proc. Natl Acad. Sci. USA.

[11] Li W (2003). Angiotensin-converting enzyme 2 is a functional receptor for the SARS coronavirus. Nature.

[12] Hoffmann M (2020). SARS-CoV-2 cell entry depends on ACE2 and TMPRSS2 and is blocked by a clinically proven protease inhibitor. Cell.

[13] Lukassen S (2020). SARS-CoV-2 receptor ACE2 and TMPRSS2 are primarily expressed in bronchial transient secretory cells. EMBO J..

[14] Sungnak W (2020). SARS-CoV-2 entry factors are highly expressed in nasal epithelial cells together with innate immune genes. Nat. Med..

[15] Han X (2020). Construction of a human cell landscape at single-cell level. Nature.

[16] Lan J (2020). Structure of the SARS-CoV-2 spike receptor-binding domain bound to the ACE2 receptor. Nature.

[17] Yan R (2020). Structural basis for the recognition of SARS-CoV-2 by full-length human ACE2. Science.

[18] Brouwer PJM (2020). Potent neutralizing antibodies from COVID-19 patients define multiple targets of vulnerability. Science.

[19] Chi X (2020). A neutralizing human antibody binds to the N-terminal domain of the Spike protein of SARS-CoV-2. Science.

[20] Mellacheruvu D (2013). The CRAPome: a contaminant repository for affinity purification-mass spectrometry data. Nat. Methods.

[21] Li X (2017). Proteomic analysis of the human tankyrase protein interaction network reveals its role in pexophagy. Cell Rep..

[22] Li X (2016). Defining the protein-protein interaction network of the human protein tyrosine phosphatase family. Mol. Cell. Proteomics.

[23] Dominguez C, Boelens R, Bonvin AMJJ (2003). HADDOCK: a protein−protein docking approach based on biochemical or biophysical information. J. Am. Chem. Soc..

[24] Munster VJ (2020). Respiratory disease in rhesus macaques inoculated with SARS-CoV-2. Nature.

[25] Shan C (2020). Infection with novel coronavirus (SARS-CoV-2) causes pneumonia in Rhesus macaques. Cell Res..

[26] Shi J (2020). Susceptibility of ferrets, cats, dogs, and other domesticated animals to SARS–coronavirus 2. Science.

[27] Sia SF (2020). Pathogenesis and transmission of SARS-CoV-2 in golden hamsters. Nature.

[28] O’Bryan JP (1991). axl, a transforming gene isolated from primary human myeloid leukemia cells, encodes a novel receptor tyrosine kinase. Mol. Cell. Biol..

[29] Goruppi, S., Ruaro, E. & Schneider, C. Gas6, the ligand of Axl tyrosine kinase receptor, has mitogenic and survival activities for serum starved NIH3T3 fibroblasts. *Oncogene* **12**, 471–480 (1996). 8637702

[30] Stitt TN (1995). The anticoagulation factor protein S and its relative, Gas6, are ligands for the Tyro 3/Axl family of receptor tyrosine kinases. Cell.

[31] Ohashi K (1995). Stimulation of sky receptor tyrosine kinase by the product of growth arrest-specific gene 6. J. Biol. Chem..

[32] Lu Q, Lemke G (2001). Homeostatic regulation of the immune system by receptor tyrosine kinases of the Tyro 3 family. Science.

[33] Morizono K (2011). The soluble serum protein Gas6 bridges virion envelope phosphatidylserine to the TAM receptor tyrosine kinase Axl to mediate viral entry. Cell Host Microbe.

[34] Monteil V (2020). Inhibition of SARS-CoV-2 Infections in engineered human tissues using clinical-grade soluble human ACE2. Cell.

[35] Harcourt J (2020). Severe acute respiratory syndrome coronavirus 2 from patient with coronavirus disease, United States. Emerg. Infect. Dis..

[36] Liao M (2020). Single-cell landscape of bronchoalveolar immune cells in patients with COVID-19. Nat. Med..

[37] Jemielity S (2013). TIM-family proteins promote infection of multiple enveloped viruses through virion-associated phosphatidylserine. PLoS Pathog..

[38] Amara A, Mercer J (2015). Viral apoptotic mimicry. Nat. Rev. Microbiol..

[39] Moller-Tank S, Kondratowicz AS, Davey RA, Rennert PD, Maury W (2013). Role of the phosphatidylserine receptor TIM-1 in enveloped-virus entry. J. Virol..

[40] Thépaut, M. et al. DC/L-SIGN recognition of spike glycoprotein promotes SARS-CoV-2 trans-infection and can be inhibited by a glycomimetic antagonist. *bioRxiv*10.1101/2020.08.09.242917 (2020). 10.1371/journal.ppat.1009576 PMC8136665 34015061

[41] Gao, C. et al. SARS-CoV-2 spike protein interacts with multiple innate immune receptors. *bioRxiv*10.1101/2020.07.29.227462 (2020).

[42] Clausen TM (2020). SARS-CoV-2 infection depends on cellular heparan sulfate and ACE2. Cell.

[43] Zhang, Q. et al. Heparan sulfate assists SARS-CoV-2 in cell entry and can be targeted by approved drugs in vitro. *bioRxiv*10.1101/2020.07.14.202549 (2020). 10.1038/s41421-020-00222-5 PMC7610239 33298900

[44] Cantuti-Castelvetri L (2020). Neuropilin-1 facilitates SARS-CoV-2 cell entry and infectivity. Science.

[45] Daly JL (2020). Neuropilin-1 is a host factor for SARS-CoV-2 infection. Science.

[46] Gu, Y. et al. Interaction network of SARS-CoV-2 with host receptome through spike protein. *bioRxiv*10.1101/2020.09.09.287508 (2020).

[47] Wei, J. et al. Genome-wide CRISPR screen reveals host genes that regulate SARS-CoV-2 infection. *bioRxiv*10.1101/2020.06.16.155101 (2020).

[48] Wang, R. et al. Functional genomic screens identify human host factors for SARS-CoV-2 and common cold coronaviruses. *bioRxiv*10.1101/2020.09.24.312298 (2020).

[49] Daniloski, Z. et al. Identification of required host factors for SARS-CoV-2 infection in human cells. *Cell*10.1016/j.cell.2020.10.030 (2020). 10.1016/j.cell.2020.10.030 PMC7584921 33147445

[50] Wang W (2014). Defining the protein-protein interaction network of the human hippo pathway. Mol. Cell. Proteomics.

[51] Shevchenko A, Wilm M, Vorm O, Mann M (1996). Mass spectrometric sequencing of proteins silver-stained polyacrylamide gels. Anal. Chem..

[52] Elias JE, Gygi SP (2007). Target-decoy search strategy for increased confidence in large-scale protein identifications by mass spectrometry. Nat. Methods.

[53] Nesvizhskii AI, Aebersold R (2005). Interpretation of shotgun proteomic data: the protein inference problem. Mol. Cell. Proteomics.

[54] Stuart T (2019). Comprehensive Integration of single-cell data. Cell.

[55] Brooks BR (2009). CHARMM: the biomolecular simulation program. J.Comput. Chem..

[56] Huang J (2017). CHARMM36m: an improved force field for folded and intrinsically disordered proteins. Nat. Methods.

[57] Eastman P (2017). OpenMM 7: rapid development of high performance algorithms for molecular dynamics. PLOS Comput. Biol..

[58] Ou X (2020). Characterization of spike glycoprotein of SARS-CoV-2 on virus entry and its immune cross-reactivity with SARS-CoV. Nat. Commun..

[59] Giraldo MI (2020). Envelope protein ubiquitination drives entry and pathogenesis of Zika virus. Nature.

[60] Zhang W (2020). Marine medaka heat shock protein 90ab1 is a receptor for red-spotted grouper nervous necrosis virus and promotes virus internalization through clathrin-mediated endocytosis. PLoS Pathog..

