# Supplementary material for: AXL is a candidate receptor for SARS-CoV-2 that promotes infection of pulmonary and bronchial epithelial cells
Source: Cell Res. 2021 Jan 8;31(2):126–40. doi: 10.1038/s41422-020-00460-y (PMC7791157; doi:10.1038/s41422-020-00460-y)
Supplement: Supplementary file 1 — Supplementary information, Fig. S1 [file 41422_2020_460_MOESM1_ESM.pdf]

Supplementary information, Fig. S1

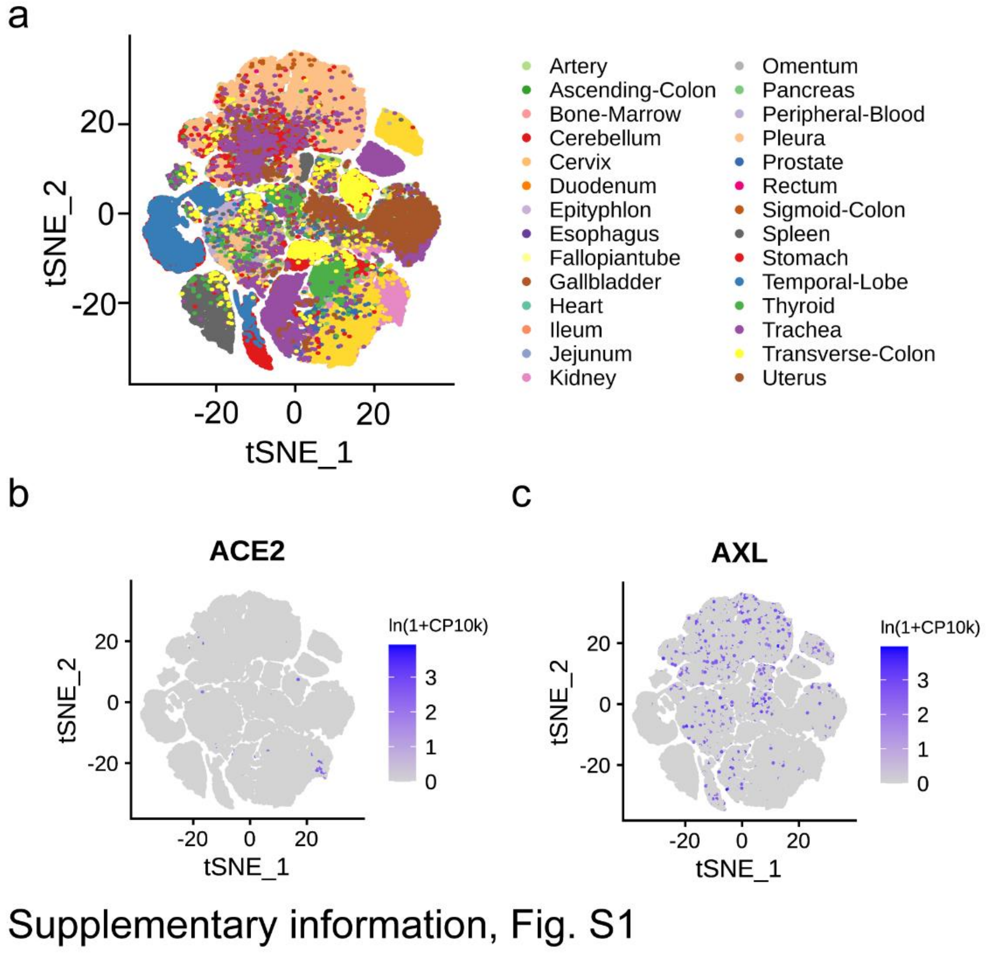

Supplementary information, Fig. S1 ACE2 and AXL expression in human organs at the 8 single-cell level. **a** An organ-specific overview of the human cell landscape at the single-cell level. **b, c** ACE2 is mainly expressed in kidney-derived cells, whereas AXL is broadly expressed in many cell types. (b) ACE2 and (c) AXL expression levels were evaluated using the human cell landscape at the single-cell level. Gene expression for each cell type in each organ was visualized using tSNE.
