# Supplementary material for: AXL is a candidate receptor for SARS-CoV-2 that promotes infection of pulmonary and bronchial epithelial cells
Source: Cell Res. 2021 Jan 8;31(2):126–40. doi: 10.1038/s41422-020-00460-y (PMC7791157; doi:10.1038/s41422-020-00460-y)
Supplement: Supplementary file 2 — Supplementary information, Fig. S2 [file 41422_2020_460_MOESM2_ESM.pdf]

Supplementary information, Fig. S2

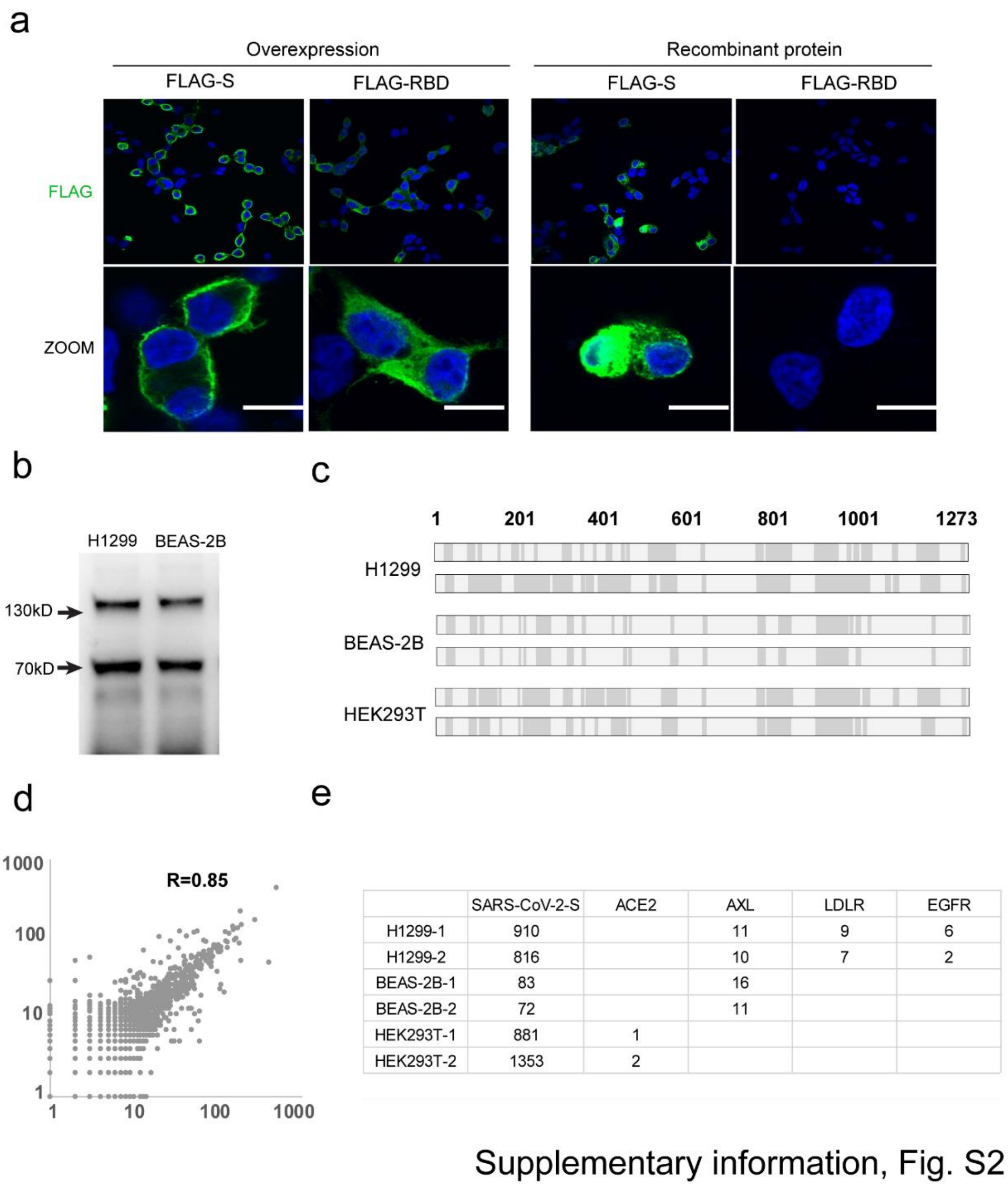

	SARS-CoV-2-S	ACE2	AXL	LDLR	EGFR
H1299-1	910		11	9	6
H1299-2	816		10	7	2
BEAS-2B-1	83		16		
BEAS-2B-2	72		11		
HEK293T-1	881	1			
HEK293T-2	1353	2			

Supplementary information, Fig. S2 Evaluation of TAP-MS methods and quality for the SARS-CoV-2 S interaction network. a Cells overexpressing full-length SARS-CoV-2 S showed strong membrane localization of the S protein. Left panel: H1299 cells were transfected with FLAG-tagged full-length SARS-CoV-2 S or its RBD for 24 h. Right panel: H1299 cells

were co-incubated with recombinant FLAG-tagged full-length SARS-CoV-2 S or its RBD for 4 h. The cells were subjected to immunofluorescence with an anti-FLAG antibody against SARS-CoV-2 S (green) and with DAPI (blue) and visualized by microscopy ($n = 3$). The scale bar indicates 15 μm. **b** Full-length SARS-CoV-2 S was successfully cleaved, generating fragments with sizes similar to those of S1-cleaved SARS-CoV-2 S. H1299 and BEAS-2B cells were transfected with FLAG-tagged full-length SARS-CoV-2 S. Expression was evaluated by Western blotting with antibodies recognizing the FLAG epitope tag ($n = 3$). **c** Bait protein coverage of SARS-CoV-2 S as determined by TAP-MS-based analyses. A schematic of the bait protein coverage in our TAP-MS-based analyses performed in the indicated cells is shown. Grey: high-confidence peptides. **d** The data reproducibility for the two biological repeats of TAP-MS analyses in each cell line was analyzed. **e** The TAP-MS results for SARS-CoV-2 S in H1299, BEAS-2B and HEK293T cells are shown in a table.
