# Supplementary material for: AXL is a candidate receptor for SARS-CoV-2 that promotes infection of pulmonary and bronchial epithelial cells
Source: Cell Res. 2021 Jan 8;31(2):126–40. doi: 10.1038/s41422-020-00460-y (PMC7791157; doi:10.1038/s41422-020-00460-y)
Supplement: Supplementary file 3 — Supplementary information, Fig. S3 [file 41422_2020_460_MOESM3_ESM.pdf]

Supplementary information, Fig. S3

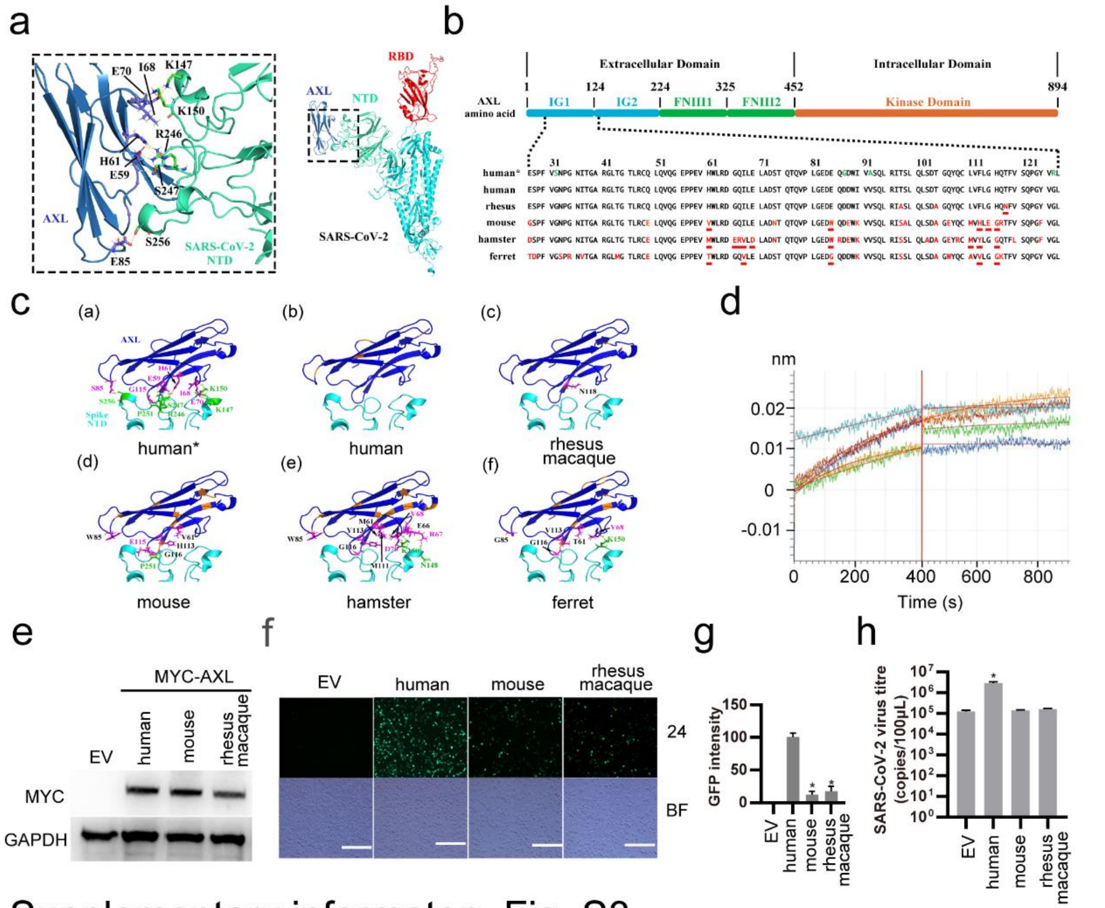

Supplementary informaton, Fig. S3

Supplementary information, Fig. S3 Human but not murine AXL interacted with the SARS-CoV-2 NTD. a Human AXL interacted with the SARS-CoV-2 NTD. Hydrogen bond interaction pairs were observed between E70 AXL and K147/K150 S NTD, I68 AXL and K150 S NTD, H61/E59 AXL and S247 S NTD, E59 AXL and R246 S NTD, and E85 AXL and S256 S NTD. There were also some hydrophobic residues at the interface, including P57-58, I68, and F113 from AXL and W152 and P251 from the S NTD, contributing to AXL binding to the S NTD. **b** AXL sequence alignment. Compared to the human AXL amino acid sequence from the

UniProt database, AXL from Protein Data Bank (PDB) entry 4RA0 (human*), whose structure was used in docking, had four mutants (green). The mutation sites in mice, hamsters and ferrets are marked in red and underlined on the AXL-S interface. The structure of AXL used for docking analysis with the S protein was obtained from PDB entry 4RA0, which bound with Gas6. Four mutations in AXL were introduced to obtain a high-affinity AXL variant. The four sites were far away from the AXL-S interface and would not change the predicted pose. **c** Locations of AXL mutation sites. Residue pairs forming hydrogen bonds at the interface of the predicted AXL-S complex are shown in (a). Different sites between the human* and human structures are in orange in (b). In (c-f), mutations at the interface are represented in magenta in the stick model, and hydrogen bonds formed with S NTD residues (green) are labeled with magenta residue index values. The mutation sites in other regions are in orange. In the predicted AXL-S complex, there were some hydrogen bonds along the interface. AXL residues E59, H61, I68, E70, S85 and G115 formed hydrogen bonds with S NTD residues S256, P251, R246, S247, K150 and K147, respectively. The four mutations in AXL from PDB entry 4RA0 were far from the interface. Only one mutation was on the interface in the rhesus macaques. Mutations at S85 and H61 in mice, hamsters and ferrets caused hydrogen bonds to disappear. Because S P251 and K150 formed hydrogen bonds with the backbone atoms of AXL G115 and I68, mutations at G115 and I68 had no impact on the hydrogen bonds. The mutation Q67R in hamsters was found to form a new hydrogen bond on the interface. Analyses of the locations of mutations and their effects on interfacial hydrogen bonds indicated that AXL binding with S protein was weakest in mice among the four species. The binding affinities of the AXL-S complexes quantitatively estimated from MM/PBSA calculations were -66.5, -46.1, -39.5, -51.6 and -44.6

kcal/mol for humans, rhesus macaques, mice, hamsters and ferrets, respectively. **d** In vitro binding assay of SARS-CoV-2 S NTD and murine AXL. His-tagged SARS-CoV-2 S NTD and FLAG-tagged murine AXL were expressed in 293F cells, affinity-purified and eluted. The KD between His-tagged SARS-CoV-2 S NTD and FLAG-tagged AXL was measured using a BLI quantification assay. e HEK293T cells were transfected with MYC-tagged human AXL, murine AXL or rhesus macaque AXL, and expression was evaluated by Western blotting with antibodies recognizing the MYC epitope tag. **f** HEK293T cells were transfected with MYC-tagged human, murine or rhesus macaque AXL for 24 h, infected with a GFP-labeled SARS-CoV-2 virus pseudotype and visualized by microscopy at 24 h post infection. The scale bar indicates 250 μm. **g** The fluorescence intensities in (f) were quantitated as indicated. The data are shown as the mean ± SEM from three independent experiments. **h** HEK293T cells stably expressing empty vector, human AXL, mouse AXL or rhesus macaque AXL were infected with SARS-CoV-2, and SARS-CoV-2 progeny titers were measured in the cell supernatants by RT-qPCR at 72 h post infection. The data shown are representative results from three independent experiments (e-h, $n = 3$). The data are shown as the mean ± SEM from three independent experiments. P values were calculated using two-way ANOVA (* $p < 0.05$, ns. not significant).
