# Supplementary material for: AXL is a candidate receptor for SARS-CoV-2 that promotes infection of pulmonary and bronchial epithelial cells
Source: Cell Res. 2021 Jan 8;31(2):126–40. doi: 10.1038/s41422-020-00460-y (PMC7791157; doi:10.1038/s41422-020-00460-y)
Supplement: Supplementary file 4 — Supplementary information, Fig. S4 [file 41422_2020_460_MOESM4_ESM.pdf]

Supplementary information, Fig. S4

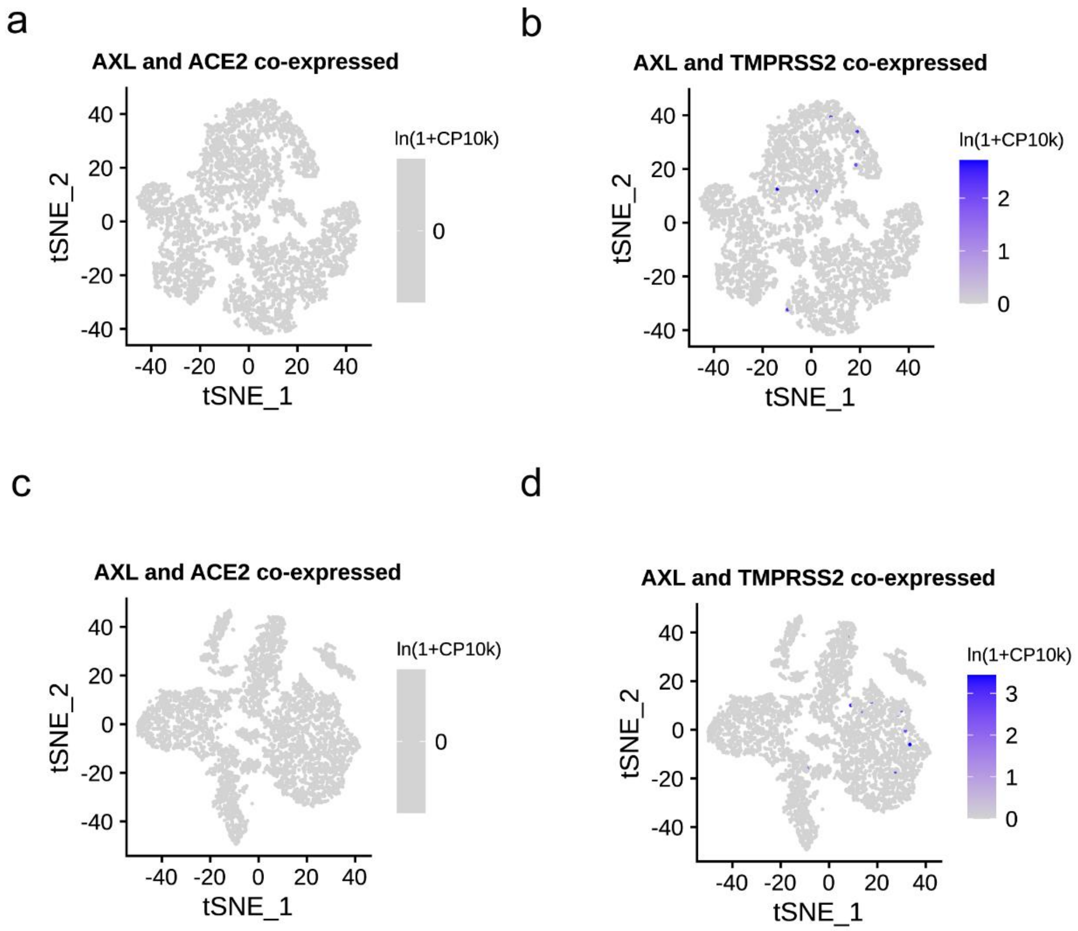

Supplementary information, Fig. S4

Supplementary information, Fig. S4 AXL is not co-expressed with ACE2 or TMPRSS2 in human lung and tracheal cells. a-d AXL and (a, c) ACE2 or TMPRSS2 (b, d) expression levels in (a, b) lung and (c, d) tracheal cells were evaluated using the human cell landscape at the single-cell level. Gene expression for each cell type was visualized using tSNE.
