# Supplementary material for: AXL is a candidate receptor for SARS-CoV-2 that promotes infection of pulmonary and bronchial epithelial cells
Source: Cell Res. 2021 Jan 8;31(2):126–40. doi: 10.1038/s41422-020-00460-y (PMC7791157; doi:10.1038/s41422-020-00460-y)
Supplement: Supplementary file 5 — Supplementary information, Fig. S5 [file 41422_2020_460_MOESM5_ESM.pdf]

Supplementary information, Fig. S5

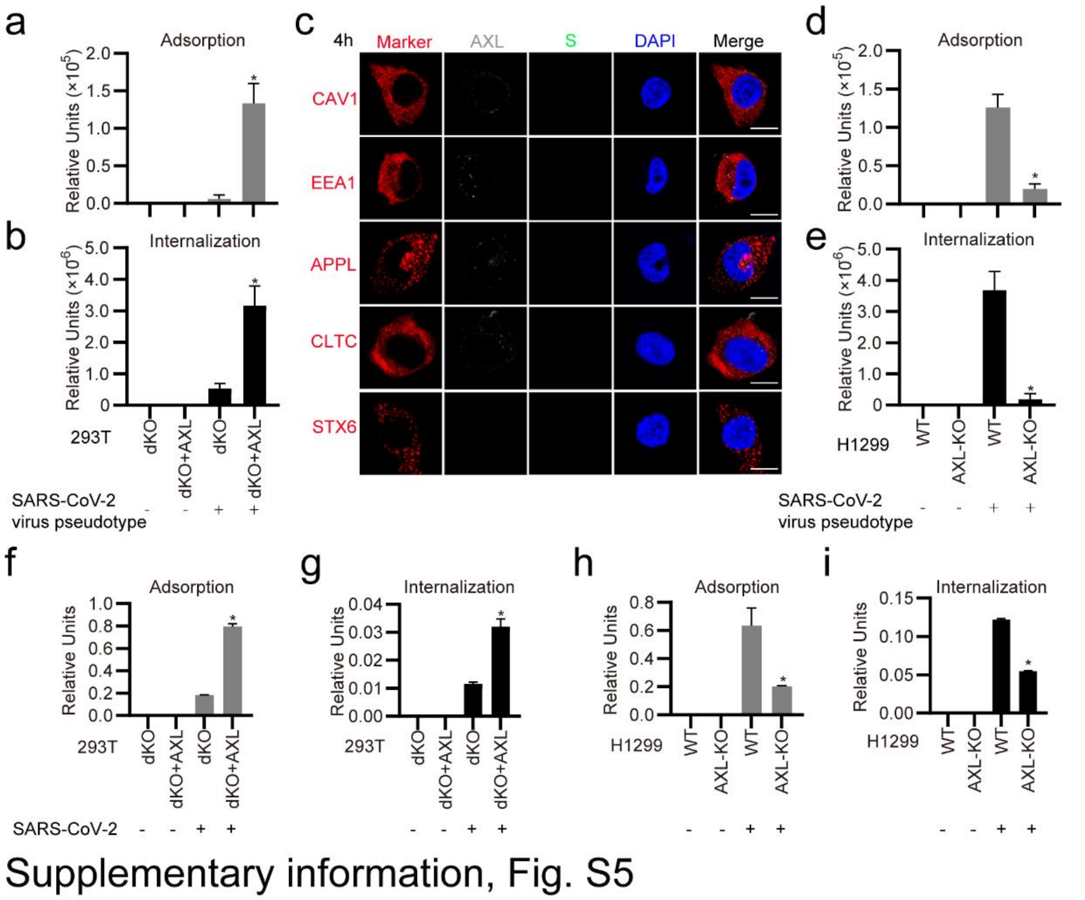

Supplementary information, Fig. S5 AXL mediates SARS-CoV-2 adsorption and internalization. a-b AXL promotes SARS-CoV-2 virus pseudotype adsorption and internalization. ACE2/AXL double-KO HEK293T cells (dKO) and ACE2/AXL double-KO HEK293T cells stably expressing AXL (dKO+AXL) were infected with vehicle or SARS-CoV-2 virus pseudotype (MOI 10) for 2 h at 4 ºC. Cells were extensively washed with PBS to remove unattached virus. **a** For binding assays, the washed cells were lysed and the expression of GFP was normalized to the expression of the GAPDH to assess the viral adsorption. **b** For internalization assays, the washed cells were incubated at 37 ºC for 2 h, then extensively washed

with PBS, followed by 0.05% trypsin-EDTA and stripping buffer to remove surface-bound virus. The cells were lysed and the expression of GFP were normalized to the expression of the GAPDH to assess the viral internalization. **c** AXL is required for SARS-CoV-2 virus pseudotype entry into host cells. H1299-AXL-KO cells were infected with a SARS-CoV-2 pseudotype for 4 h. The cells were fixed; subjected to immunofluorescence with antibodies against AXL (grey), SARS-CoV-2 S (green) and the indicated endocytosis-related proteins (red) and with DAPI (blue); and visualized by confocal microscopy. The scale bar indicates 15 μm. **d-e** Knocking out AXL inhibits SARS-CoV-2 virus pseudotype adsorption and internalization. Control or AXL-KO H1299 cells were infected with vehicle or SARS-CoV-2 virus pseudotype (MOI 10). **d** viral adsorption and **e** internalization were assessed as in (a) and (b), respectively. **f-g** AXL promotes SARS-CoV-2 adsorption and internalization. ACE2/AXL double-KO HEK293T cells and ACE2/AXL double-KO HEK293T cells stably expressing AXL were infected with vehicle or SARS-CoV-2 (MOI 10) for 2 h at 4 ºC. Cells were extensively washed with PBS to remove unattached virus. **f** For binding assays, the washed cells were lysed and the expression of the SARS-CoV-2 N gene was normalized to the level of the GAPDH to assess the viral adsorption. **g** For internalization assays, the washed cells were incubated at 37 ºC for 2 h, then extensively washed with PBS, followed by 0.05% trypsin-EDTA and stripping buffer to remove surface-bound virus. The cells were lysed and expression of the SARS-CoV-2 N gene was normalized to the level of the GAPDH to assess the viral internalization. **h-i** Knocking out AXL inhibits SARS-CoV-2 adsorption and internalization. Control or AXL-KO H1299 cells were infected with vehicle or SARS-CoV-2 (MOI 10). **h** viral adsorption and **i** internalization were assessed as in (f) and (g), respectively. The data shown are representative results from

three independent experiments (a-i, $n = 3$). The data are shown as the mean ± SEM from three independent experiments. *P* values were calculated using two-way ANOVA (* $p < 0.05$).
