# Supplementary material for: AXL is a candidate receptor for SARS-CoV-2 that promotes infection of pulmonary and bronchial epithelial cells
Source: Cell Res. 2021 Jan 8;31(2):126–40. doi: 10.1038/s41422-020-00460-y (PMC7791157; doi:10.1038/s41422-020-00460-y)
Supplement: Supplementary file 6 — Supplementary information, Fig. S6 [file 41422_2020_460_MOESM6_ESM.pdf]

Supplementary information, Fig. S6

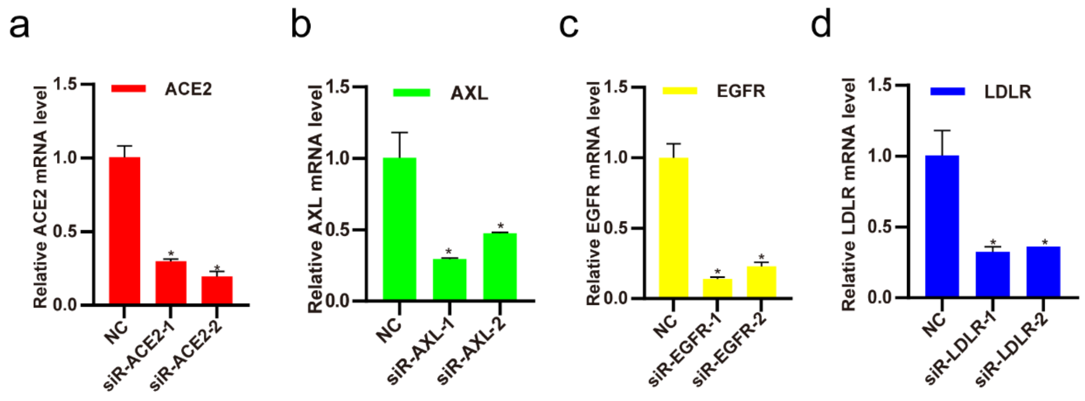

Supplementary information, Fig. S6

Supplementary information, Fig. S6 Knockdown using siRNA against ACE2 or candidate receptors. a-d H1299 cells were transfected with siRNA against (a) ACE2, (b) AXL, (c) EGFR or (d) LDLR for 24 h, and the knockdown efficiency was evaluated using RT-qPCR. The data shown are representative results from three independent experiments (n = 3). The data are shown as the mean ± SEM from three independent experiments. P values were calculated using two-way ANOVA (* $p < 0.05$).
