# Supplementary material for: AXL is a candidate receptor for SARS-CoV-2 that promotes infection of pulmonary and bronchial epithelial cells
Source: Cell Res. 2021 Jan 8;31(2):126–40. doi: 10.1038/s41422-020-00460-y (PMC7791157; doi:10.1038/s41422-020-00460-y)
Supplement: Supplementary file 7 — Supplementary information, Fig. S7 [file 41422_2020_460_MOESM7_ESM.pdf]

Supplementary information, Fig. S7

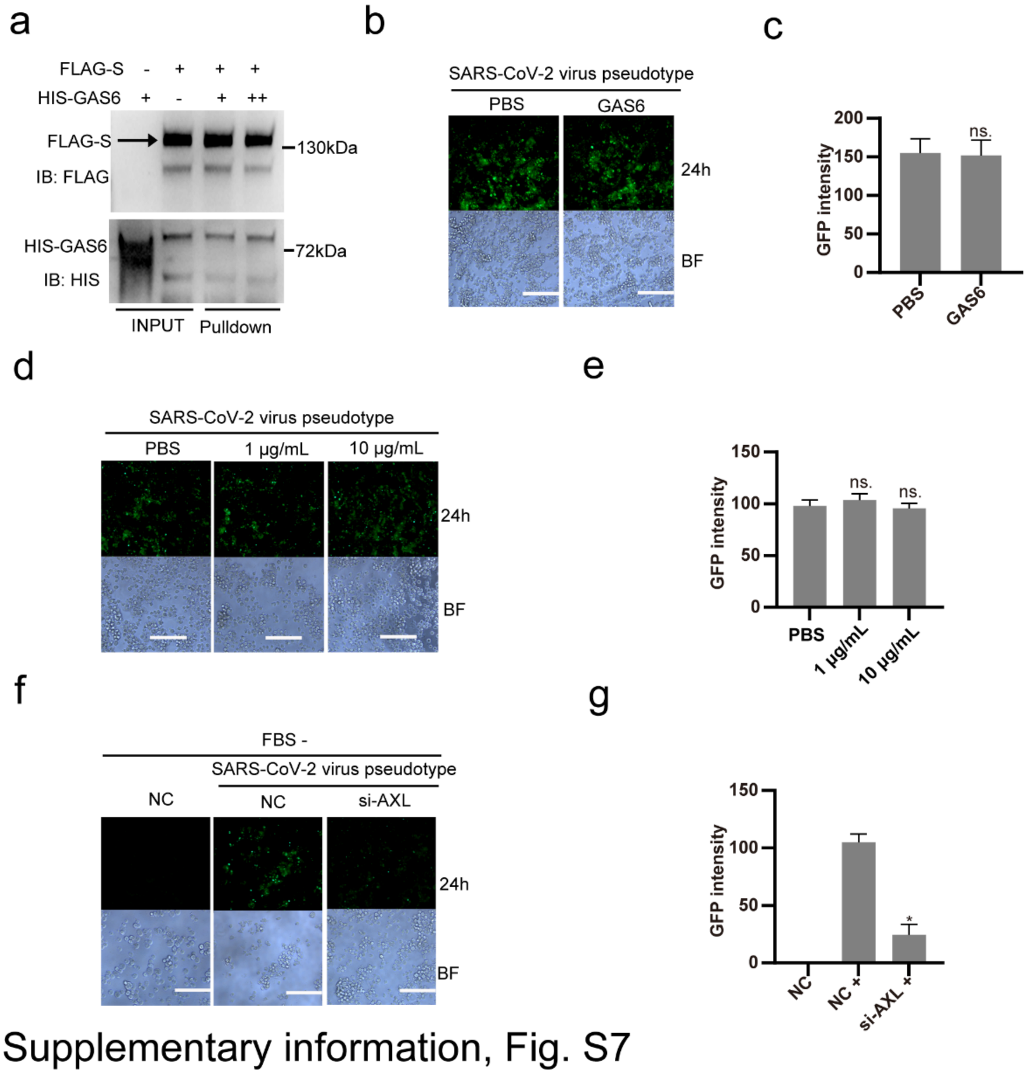

Supplementary information, Fig. S7 GAS6 and Protein S in serum are dispensable in AXL-induced SARS-CoV-2 virus pseudotype infection. a GAS6 does not bind SARS-CoV-2 S. In vitro pull-down assay of SARS-CoV-2 S and GAS6. FLAG-tagged SARS-CoV-2 S and His-tagged GAS6 were purified and co-incubated for 1 h. Blots with antibodies recognizing the FLAG- or His-epitope tags are shown. **b** GAS6 does not promote AXL-induced SARS-CoV-2 virus pseudotype infection. HEK293T cells were transfected with AXL, incubated with GAS6

recombinant protein, infected with the GFP-labeled SARS-CoV-2 virus pseudotype, and visualized by microscopy at 24 h post infection. The scale bar indicates 250 μm. **c** The fluorescence intensities in (b) were quantitated as indicated. **d** Protein S is dispensable in AXL-induced SARS-CoV-2 virus pseudotype infection. H1299 cells were maintained in serum-free medium. Cells were transfected with siRNA against AXL or control siRNA, infected with the GFP-labeled SARS-CoV-2 pseudotype, and visualized by microscopy at 24 h post infection. The scale bar indicates 250 μm. **e** The fluorescence intensities in (d) were quantitated as indicated. **f** Soluble GAS6 protein does not promote SARS-CoV-2 virus pseudotype infection. H1299 cells were maintained in serum-free medium. Human recombinant GAS6 recombinant protein (1 or 10 μg/ml) was mixed with the SARS-CoV-2 virus pseudotype for 30 min and then added to the culture medium of H1299 cells. The cells were washed at 2 h post infection, incubated with fresh medium, and visualized by microscopy at 24 h post infection. The scale bar indicates 250 μm. **g** The fluorescence intensities in (f) were quantitated as indicated. The data shown are representative results from three independent experiments (a-g, $n = 3$). The data are shown as the mean ± SEM from three independent experiments. *P* values were calculated using two-way ANOVA (* $p < 0.05$, ns. not significant).
