# Supplementary material for: AXL is a candidate receptor for SARS-CoV-2 that promotes infection of pulmonary and bronchial epithelial cells
Source: Cell Res. 2021 Jan 8;31(2):126–40. doi: 10.1038/s41422-020-00460-y (PMC7791157; doi:10.1038/s41422-020-00460-y)
Supplement: Supplementary file 8 — Supplementary information, Table Legends [file 41422_2020_460_MOESM8_ESM.pdf]

Supplementary information, Table legends

Supplementary information, Table S1. Summary of gene expression in different human tissues at the single-cell level. The numbers of ACE2-, AXL-, and TMPRSS2-positive cells and the numbers of cells co-expressing these proteins in different human tissues were calculated using the human cell landscape at the single-cell level.

Supplementary information, Table S2. Complete protein identification list for SARS-CoV-2 and influenza A viruses. Complete protein and peptide identification lists for SARS-CoV-2 and influenza A virus (A/Guangzhou/39715/2014 (H5N6)) were collected for all the TAP- and AP-MS experiments.

Supplementary information, Table S3. Complete protein identification list for humans. The complete protein and peptide identification lists for *Homo sapiens* were collected for all the TAP-MS and AP-MS experiments.

Supplementary information, Table S4. Complete protein identification list for mFc-tagged SARS-CoV-2 S recombinant protein AP-MS in H1299 cells. mFc-tagged SARS-CoV-2 S recombinant protein was used for pull-down in H1299 cells followed by MS analyses. The complete protein and peptide identification lists for SARS-CoV-2 and *Homo sapiens* were collected for SARS-CoV-2 S TAP-MS in HEK293T cells.
